# Supplementary material for: The behavior of sympatric sea urchin species across an ecosystem state gradient
Source: PeerJ. 2023 Jun 13;11:e15511. doi: 10.7717/peerj.15511 (PMC10274604; doi:10.7717/peerj.15511)
Supplement: Supplemental Information 1 — Document containing (A) the mean wind fetch for the isoyake, (B) the average daily wave heights, PPFD and temperature conditions of the vegetated and isoyake habitats, and (C) the figures for all model validations, each showing their kernel density estimates of the observations and predictions and rank plots for the MCMC draws. [file peerj-11-15511-s001.pdf]

Supplementary information

The behavior of sympatric sea urchin species across an ecosystem state gradient

Dominic Franco C. Belleza, Takeshi Urae, Shinichiro Tanimae, Kento Toyama, Akari Isoda, Gregory N. Nishihara

I. Wind fetch

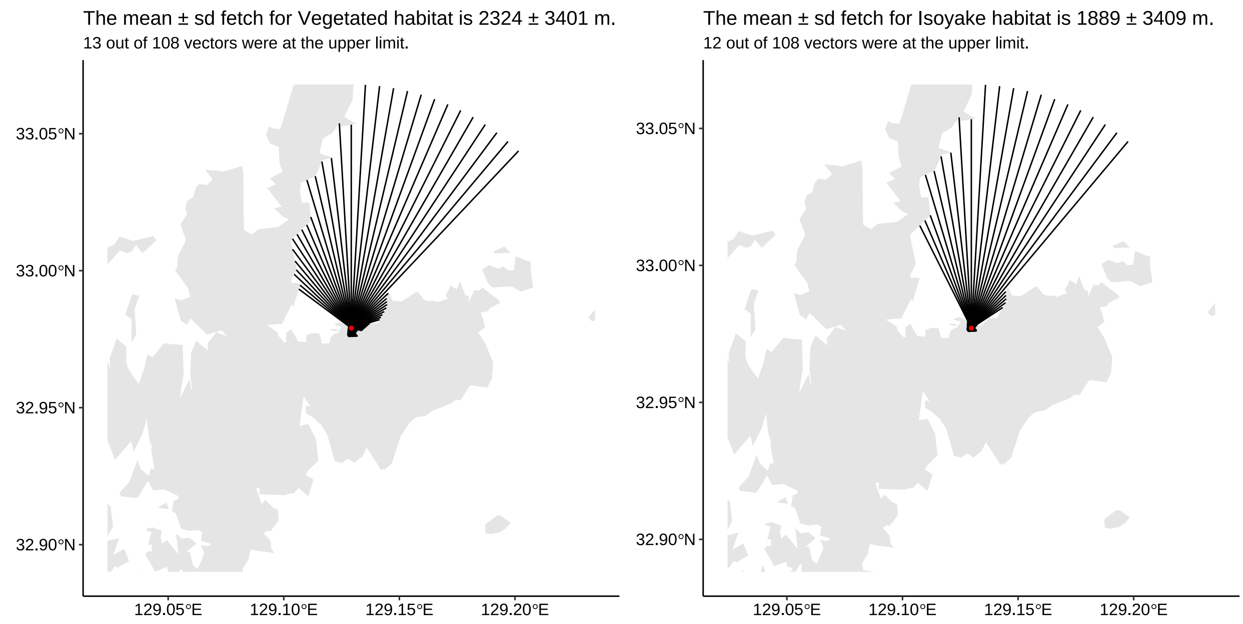

Figure S1. The mean fetch of the Vegetated and Isoyake habitats based on 108 vectors. The lines are the wind fetch vectors while the red points are the coordinates from the study sites.

II. Average daily wave heights, PPFD and temperature

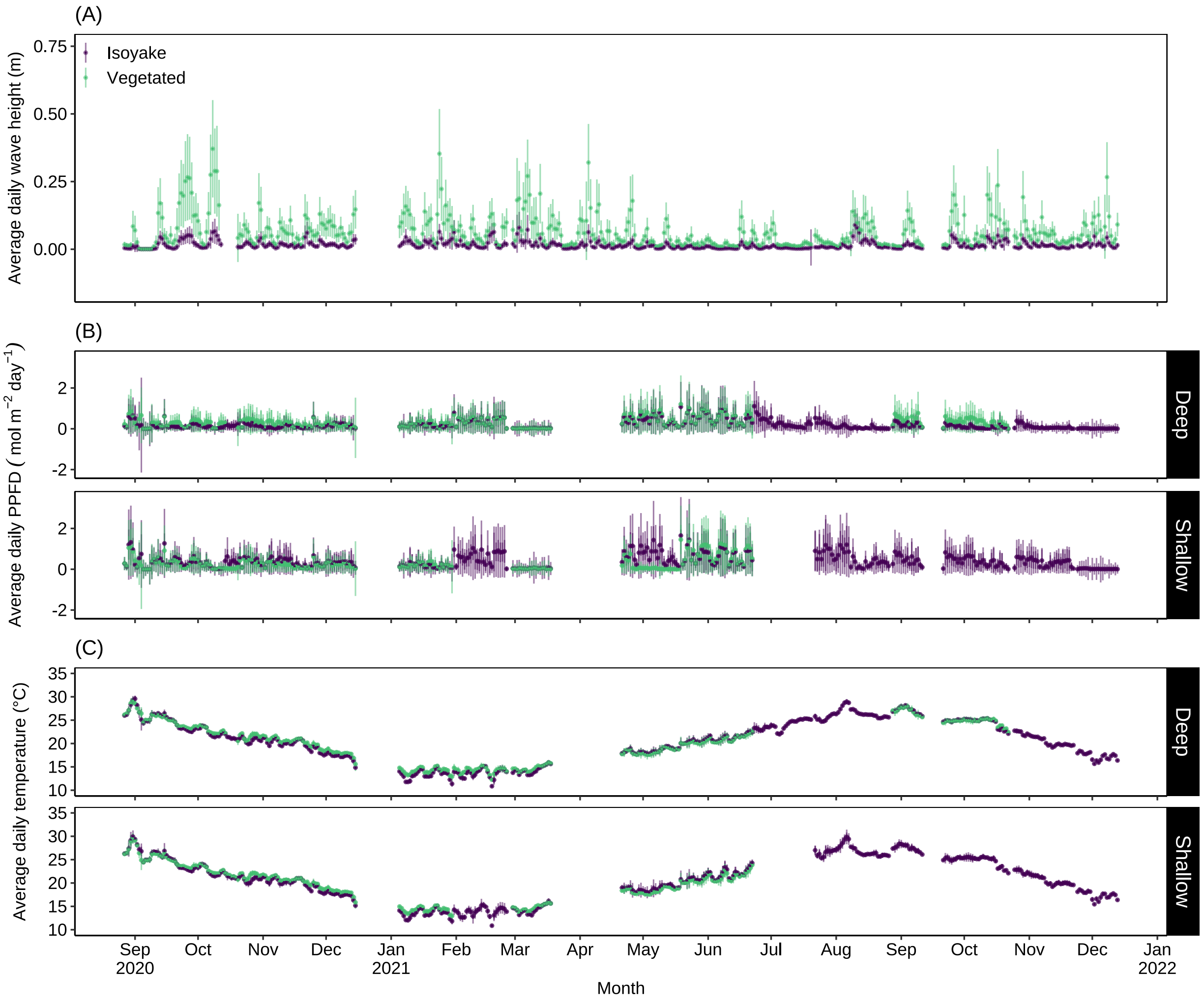

Figure S2. The average daily (A) wave heights, (B) photosynthetic photon flux density (PPFD), and (C) temperature of the study sites. The points are the mean values while the vertical bars are the standard deviations.

III. Environmental data model validation

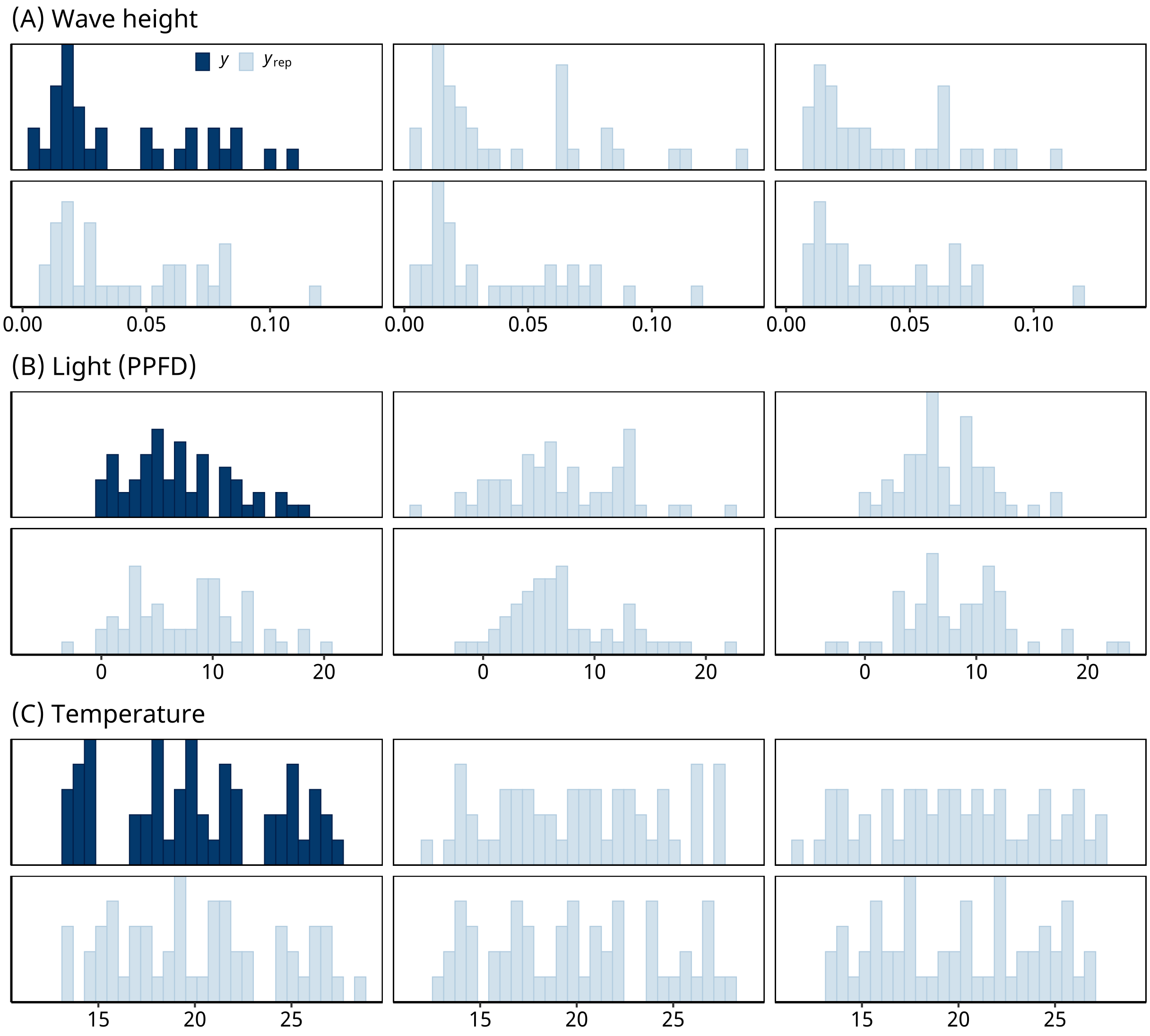

Figure S3. The kernel density estimates of the observations y and the model predictions y_{rep} from the posterior predictions of the environmental data models. The model predictions should be able to approximate the pattern of the observations and thus appear similar.

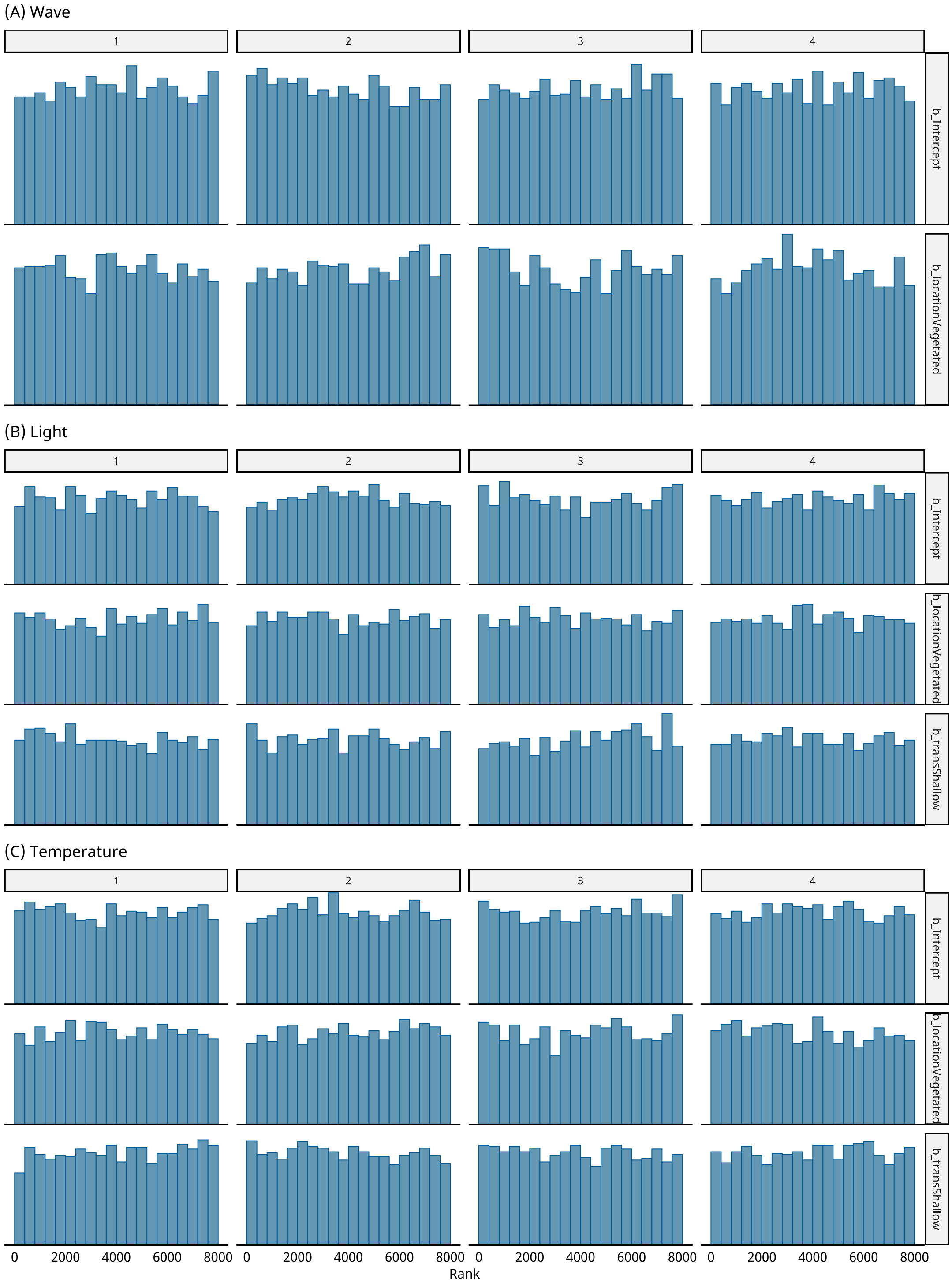

Figure S4. The rank plot of the MCMC draws for β (i.e., covariates) from the environmental data models. The columns are the chains. The bars should appear similar.

IV. Benthic rugosity model validation

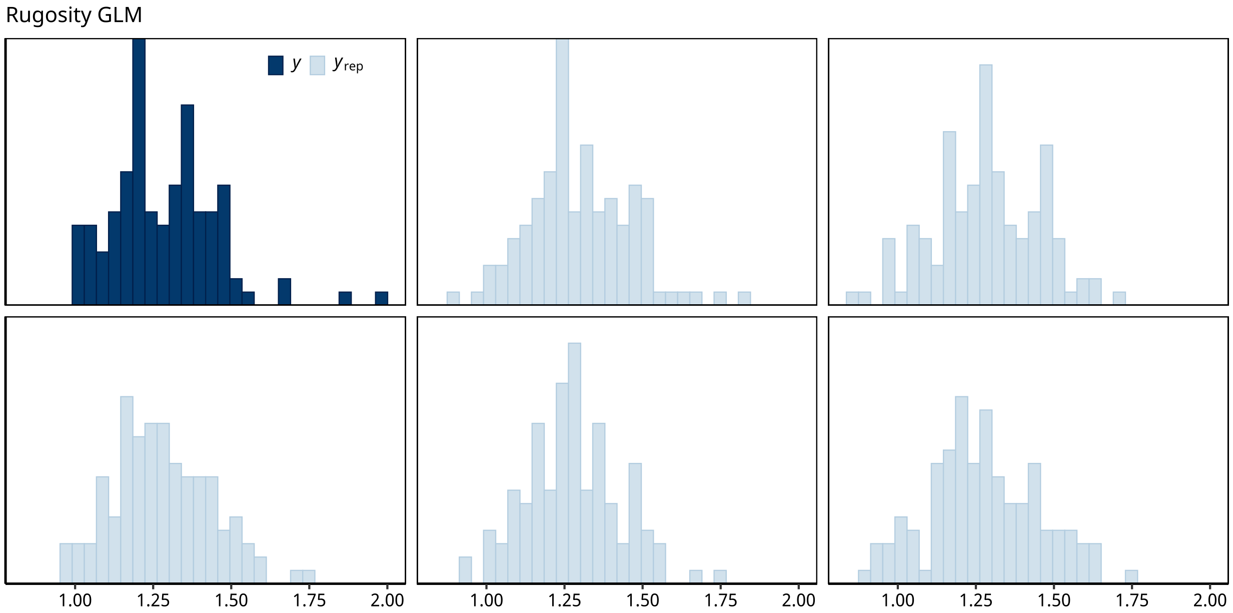

Figure S5. The kernel density estimates of the observations y and the model predictions y_{rep} from the posterior predictions of the benthic rugosity model. The model predictions should be able to approximate the pattern of the observations and thus appear similar.

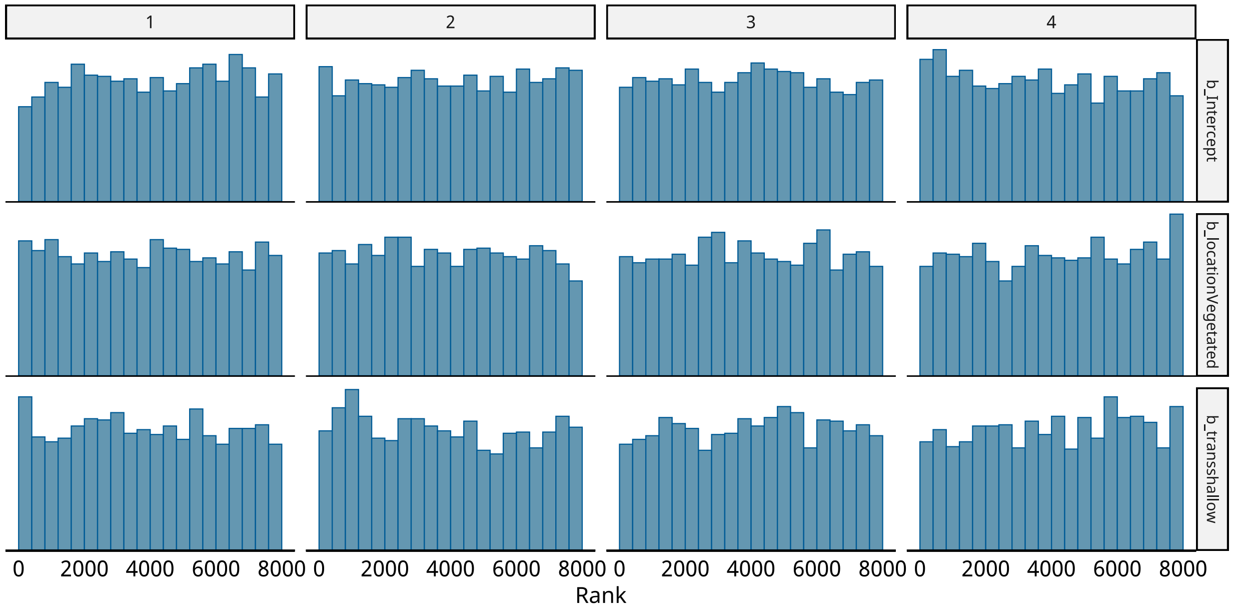

Figure S6. The rank plot of the MCMC draws for β (i.e., categorical variables) from the benthic rugosity model. The columns are the chains. The bars should appear similar.

V. Benthic cover model validation

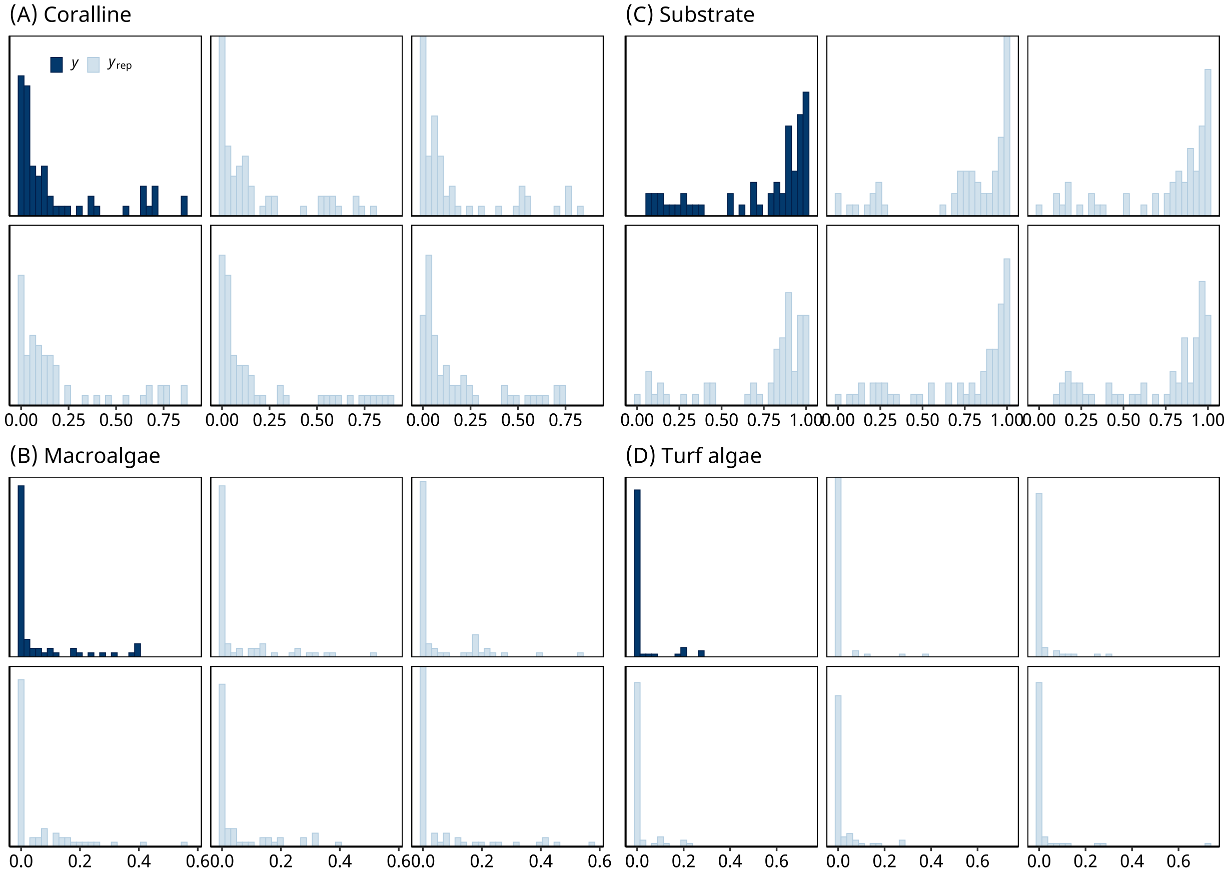

Figure S7. The kernel density estimates of the observations y and the model predictions y_{rep} from the posterior predictions of the benthic cover models. The model predictions should be able to approximate the pattern of the observations and thus appear similar.

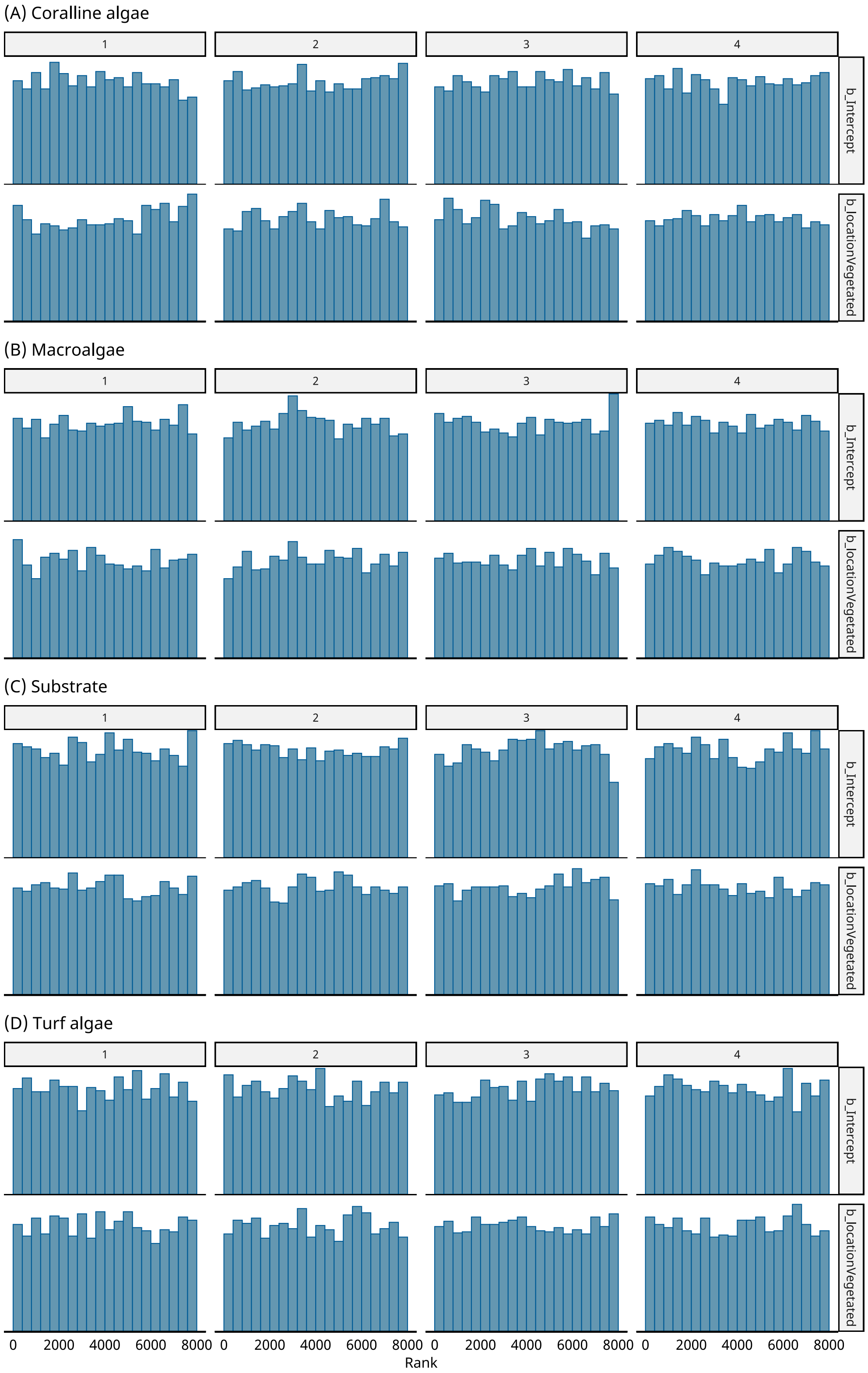

Figure S8. The rank plot of the MCMC draws for β (i.e., categorical variables) from the benthic cover models. The columns are the chains. The bars should appear similar.

VI. Urchin density and biomass model validation

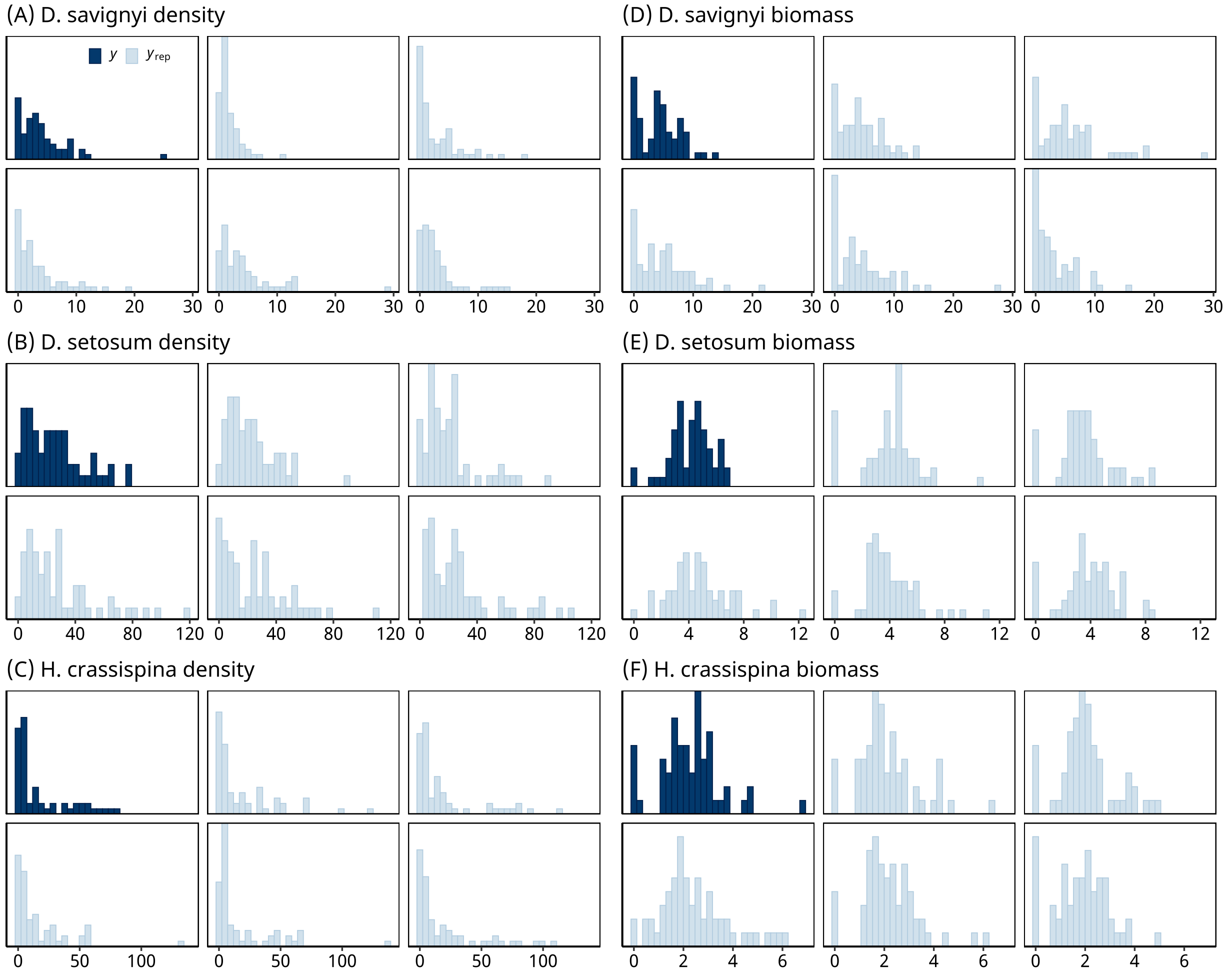

Figure S9. The kernel density estimates of the observations y and the model predictions y_{rep} from the posterior predictions of the urchin density (A-C) and urchin biomass (D-F) models. The model predictions should be able to approximate the pattern of the observations and thus appear similar.

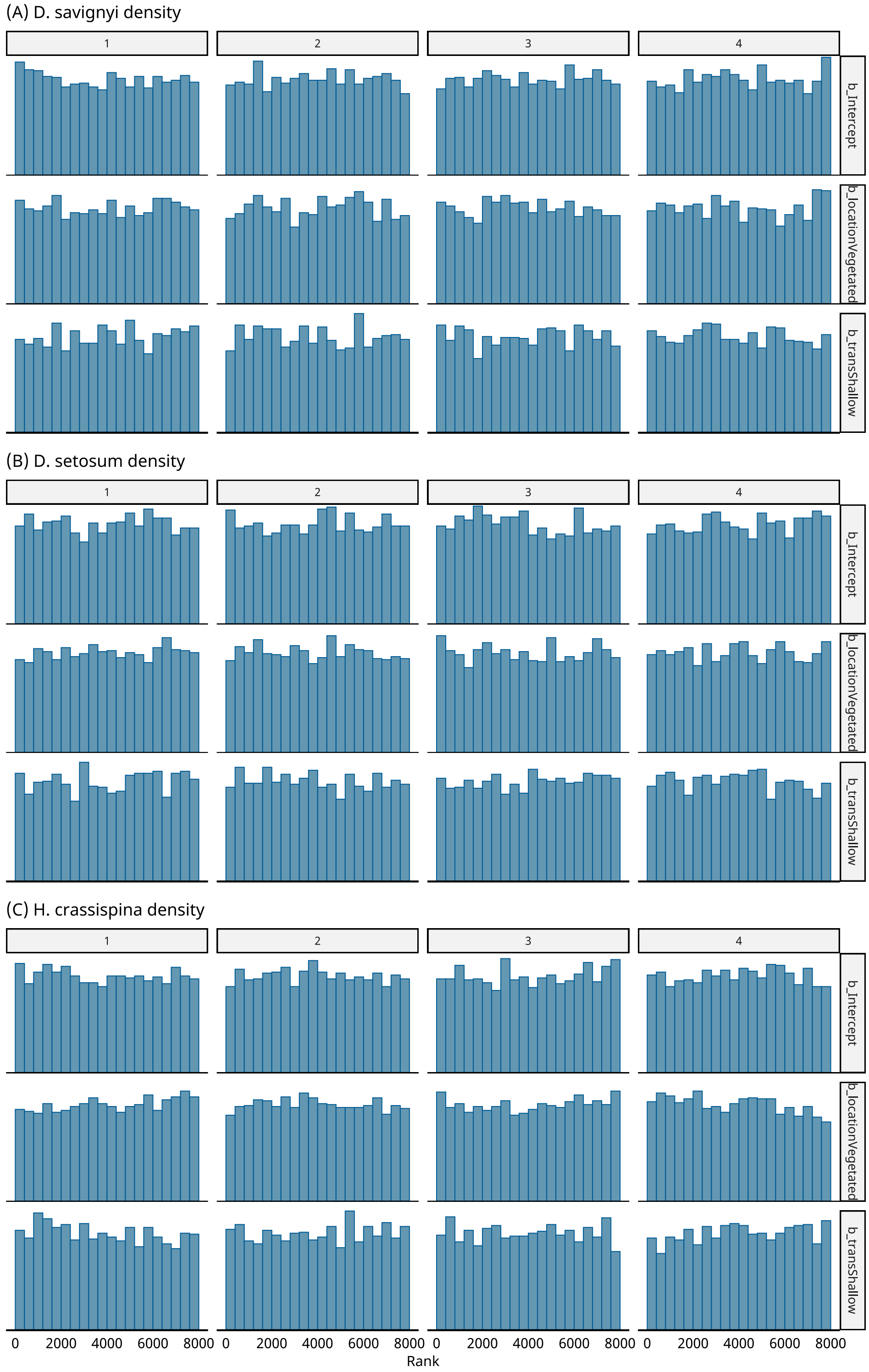

Figure S10. The rank plot of the MCMC draws for β (i.e., categorical variables) from the urchin density models. The columns are the chains. The bars should appear similar.

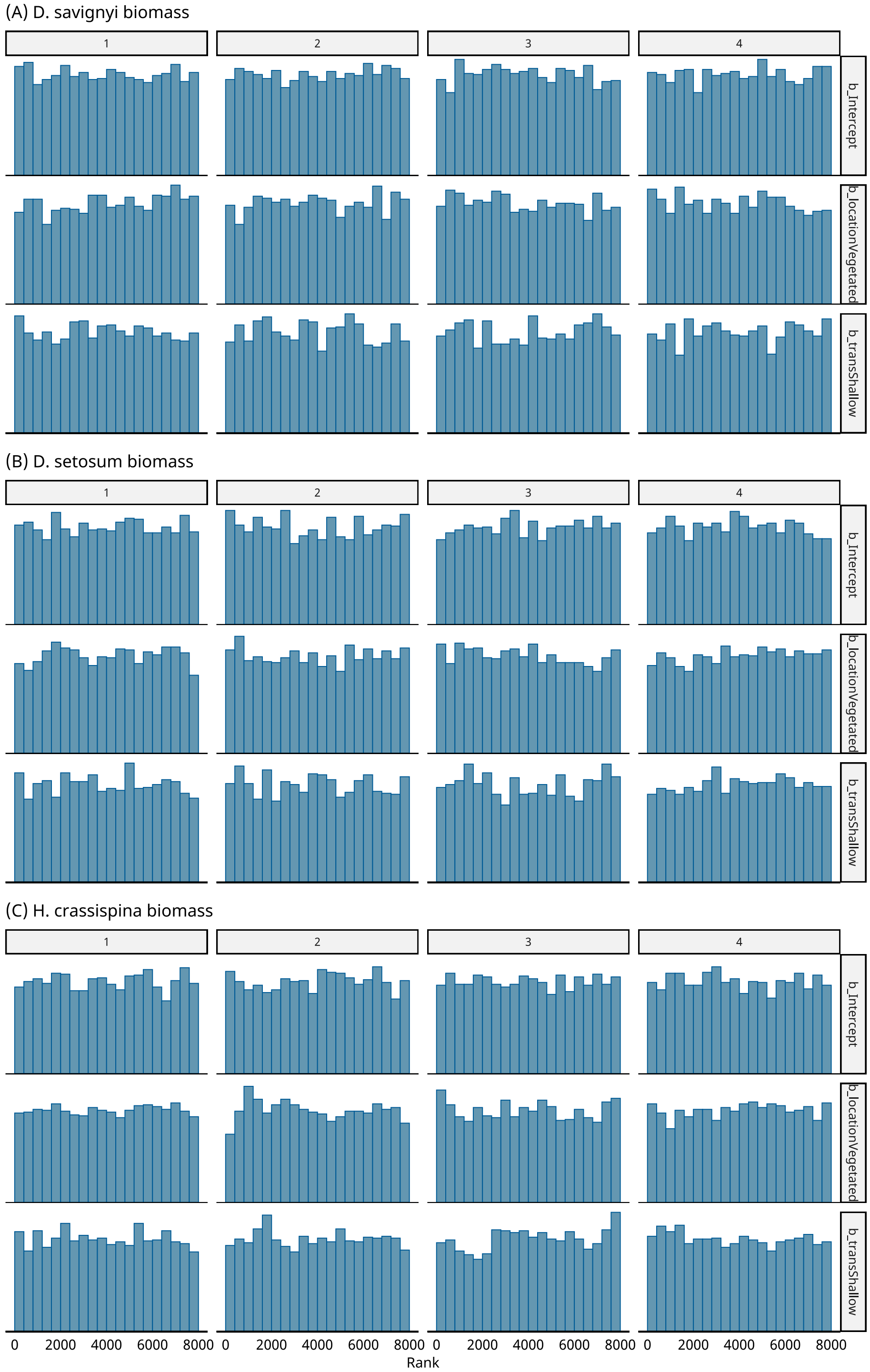

Figure S11. The rank plot of the MCMC draws for β (i.e., categorical variables) from the urchin biomass models. The columns are the chains. The bars should appear similar.

VII. Urchin microhabitat model validation

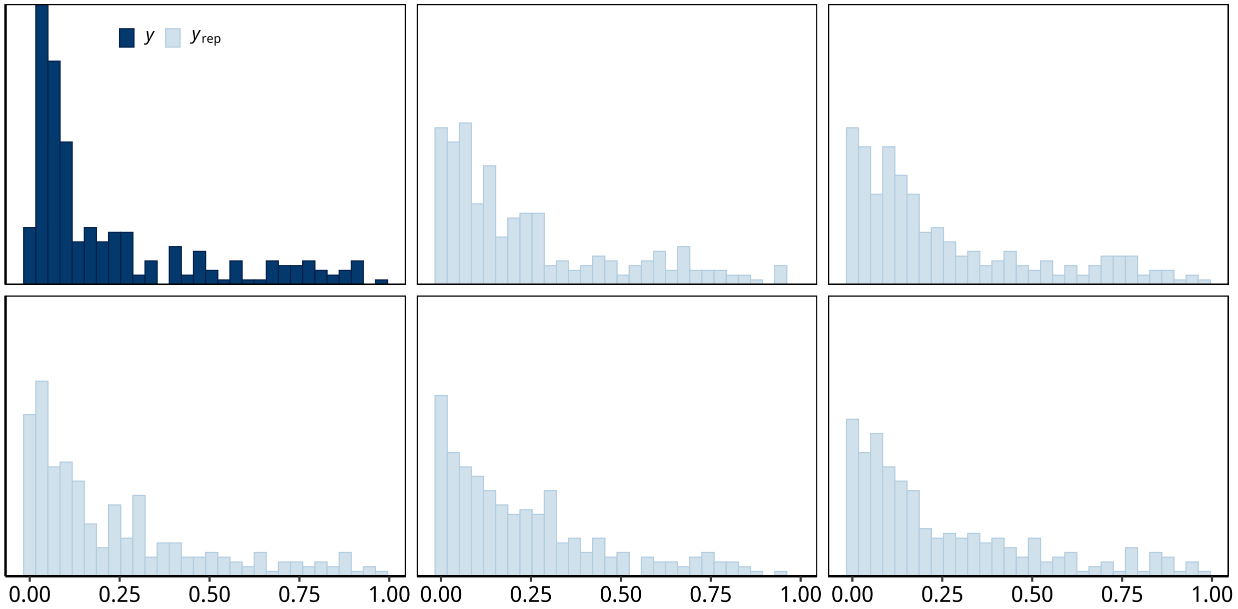

Figure S12. The kernel density estimates of the observations y and the model predictions y_{rep} from the posterior predictions of the urchin microhabitat model. The model predictions should be able to approximate the pattern of the observations and thus appear similar.

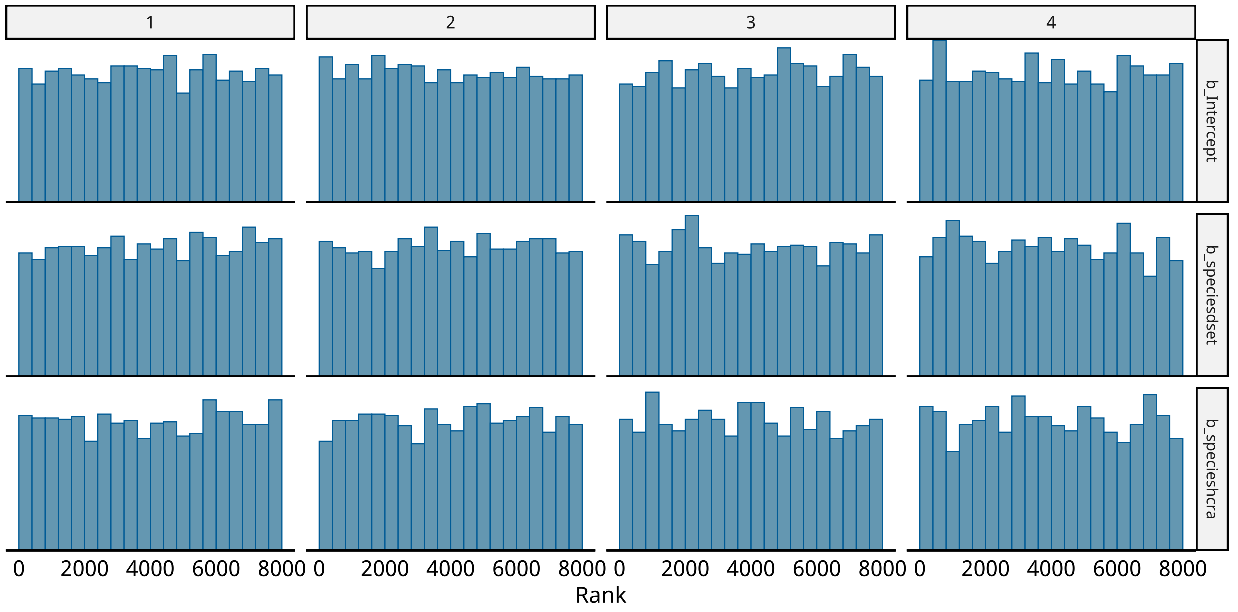

Figure S13. The rank plot of the MCMC draws for β (i.e., categorical variables) from the urchin microhabitat model. The columns are the chains. The bars should appear similar.

VIII. Linear displacement model

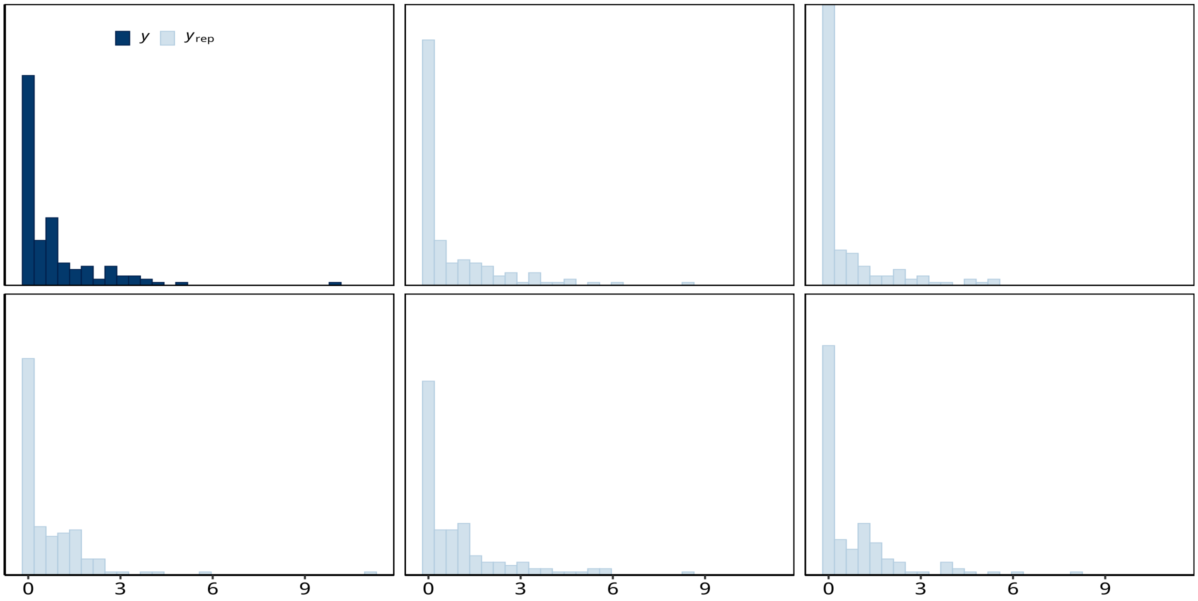

Figure S14. The kernel density estimates of the observations y and the model predictions y_{rep} from the posterior predictions of the linear distance model. The model predictions should be able to approximate the pattern of the observations and thus appear similar.

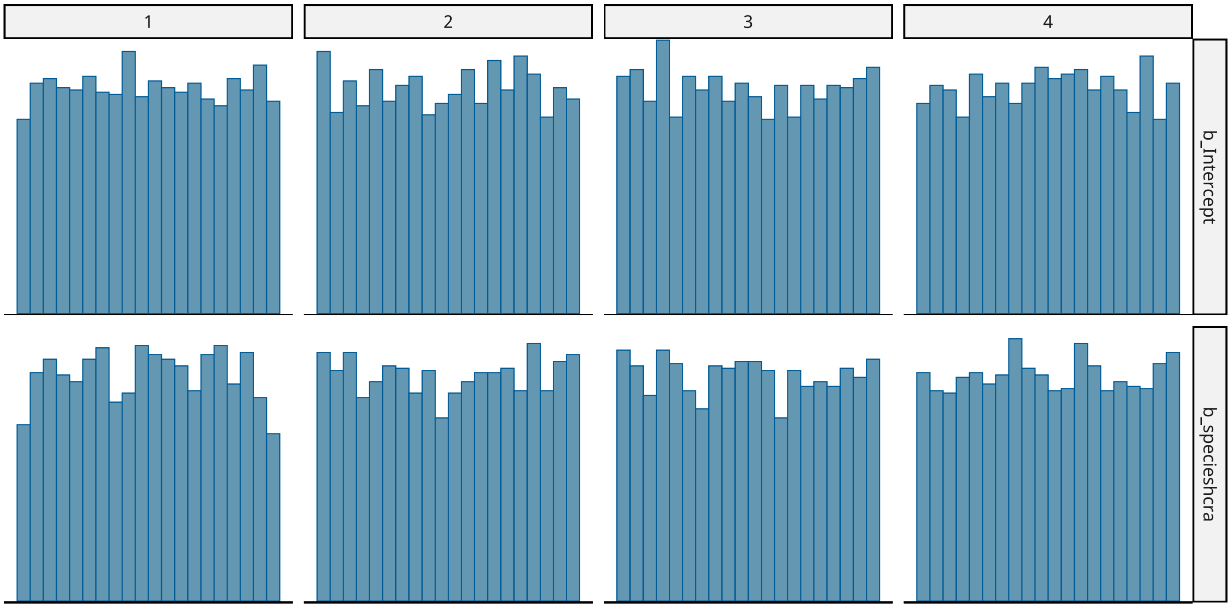

Figure S15. The rank plot of the MCMC draws for β (i.e., categorical variables) from the linear distance model. The columns are the chains. The bars should appear similar.

IX. Group-size model

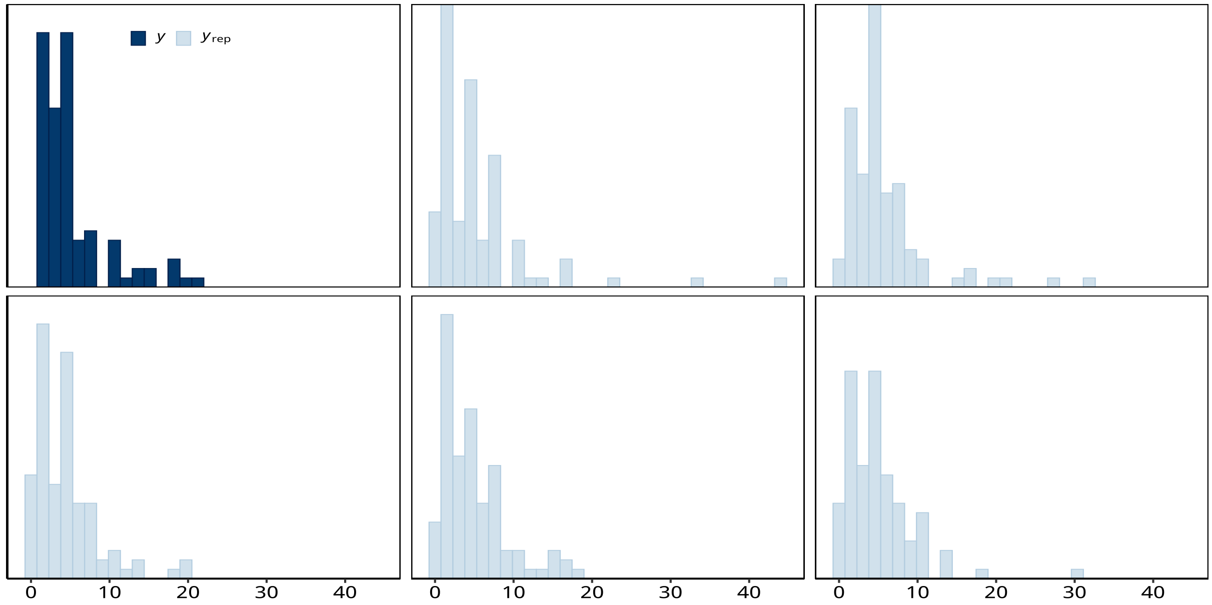

Figure S16. The kernel density estimates of the observations y and the model predictions y_{rep} from the posterior predictions of the group-size model. The model predictions should be able to approximate the pattern of the observations and thus appear similar.

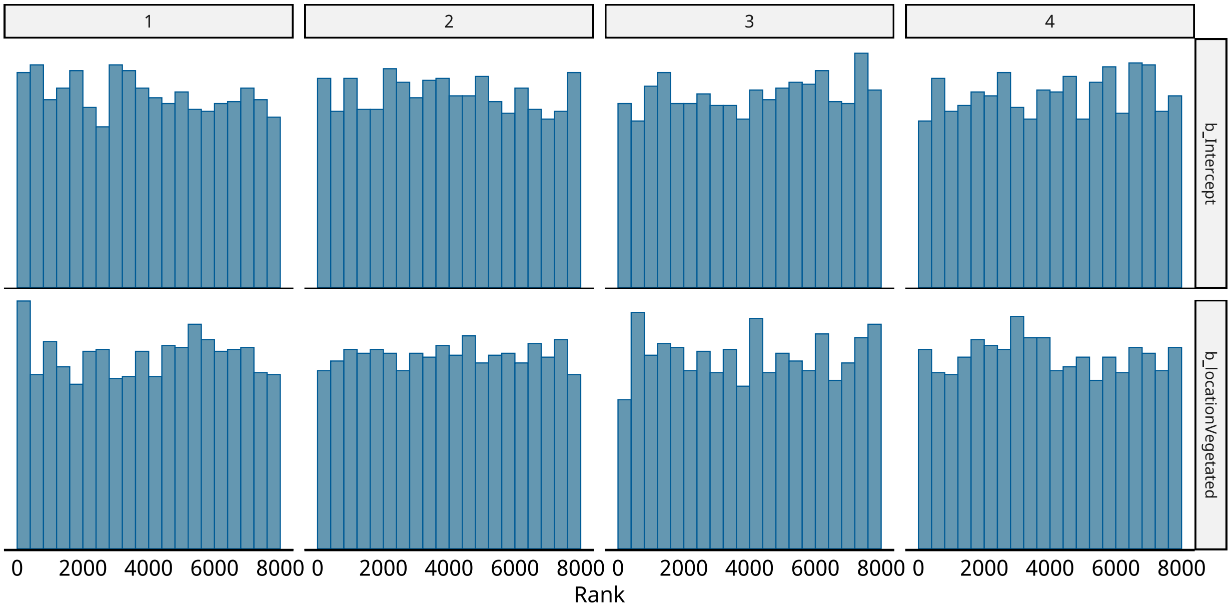

Figure S17. The rank plot of the MCMC draws for β (i.e., categorical variables) from the group-size model. The columns are the chains. The bars should appear similar.
