# Supplementary material for: The behavior of sympatric sea urchin species across an ecosystem state gradient
Source: PeerJ. 2023 Jun 13;11:e15511. doi: 10.7717/peerj.15511 (PMC10274604; doi:10.7717/peerj.15511)
Supplement: Supplemental Information 2 — The mean and 95% highest density credible interval for the expectations of the generalized additive model (GAM) of the monthly daily average wave heights (m) in the isoyake and vegetated habitat. [file peerj-11-15511-s002.docx]

| **Month** | **Habitat** | **Monthly daily average wave height (m) GAM** | | |
| --- | --- | --- | --- | --- |
|  |  | **Mean** | **Lower** | **Upper** |
| 2020-Sep | Isoyake | 0.02 | 0.01 | 0.03 |
| 2020-Oct | Isoyake | 0.02 | 0.02 | 0.03 |
| 2020-Nov | Isoyake | 0.02 | 0.01 | 0.02 |
| 2020-Dec | Isoyake | 0.02 | 0.01 | 0.02 |
| 2021-Jan | Isoyake | 0.02 | 0.02 | 0.02 |
| 2021-Feb | Isoyake | 0.02 | 0.02 | 0.03 |
| 2021-Mar | Isoyake | 0.02 | 0.01 | 0.02 |
| 2021-Apr | Isoyake | 0.01 | 0.01 | 0.02 |
| 2021-May | Isoyake | 0.01 | 0.01 | 0.01 |
| 2021-Jun | Isoyake | 0.01 | 0.01 | 0.01 |
| 2021-Jul | Isoyake | 0.01 | 0.01 | 0.01 |
| 2021-Aug | Isoyake | 0.01 | 0.01 | 0.02 |
| 2021-Sep | Isoyake | 0.02 | 0.01 | 0.02 |
| 2021-Oct | Isoyake | 0.02 | 0.01 | 0.02 |
| 2021-Nov | Isoyake | 0.02 | 0.01 | 0.02 |
| 2021-Dec | Isoyake | 0.02 | 0.01 | 0.02 |
| 2020-Sep | Vegetated | 0.09 | 0.06 | 0.12 |
| 2020-Oct | Vegetated | 0.08 | 0.06 | 0.10 |
| 2020-Nov | Vegetated | 0.08 | 0.06 | 0.09 |
| 2020-Dec | Vegetated | 0.07 | 0.06 | 0.09 |
| 2021-Jan | Vegetated | 0.08 | 0.06 | 0.10 |
| 2021-Feb | Vegetated | 0.08 | 0.06 | 0.10 |
| 2021-Mar | Vegetated | 0.07 | 0.05 | 0.09 |
| 2021-Apr | Vegetated | 0.05 | 0.04 | 0.07 |
| 2021-May | Vegetated | 0.04 | 0.03 | 0.05 |
| 2021-Jun | Vegetated | 0.03 | 0.02 | 0.04 |
| 2021-Jul | Vegetated | 0.04 | 0.03 | 0.05 |
| 2021-Aug | Vegetated | 0.05 | 0.04 | 0.06 |
| 2021-Sep | Vegetated | 0.06 | 0.05 | 0.08 |
| 2021-Oct | Vegetated | 0.07 | 0.05 | 0.09 |
| 2021-Nov | Vegetated | 0.07 | 0.06 | 0.09 |
| 2021-Dec | Vegetated | 0.07 | 0.05 | 0.10 |
