# Supplementary material for: The behavior of sympatric sea urchin species across an ecosystem state gradient
Source: PeerJ. 2023 Jun 13;11:e15511. doi: 10.7717/peerj.15511 (PMC10274604; doi:10.7717/peerj.15511)
Supplement: Supplemental Information 3 — The mean and 95% highest density credible interval for the expectations of the generalized additive model (GAM) applied to the monthly daily average photosynthetic photon flux density (PPFD, mol m−2 day−1) in the deep and shallow transects of the isoyake and vegetated habitat. [file peerj-11-15511-s003.docx]

| **Month** | **Habitat** | **Transect** | **Monthly daily average PPFD (mol m^-2^ day^-1^) GAM** | | |
| --- | --- | --- | --- | --- | --- |
|  |  |  | **Mean** | **Lower** | **Upper** |
| 2020-Sep | Isoyake | Deep | 4.18 | 2.18 | 6.32 |
| 2020-Oct | Isoyake | Deep | 3.48 | 2.04 | 4.86 |
| 2020-Nov | Isoyake | Deep | 3.05 | 1.80 | 4.27 |
| 2020-Dec | Isoyake | Deep | 2.92 | 1.66 | 4.26 |
| 2021-Jan | Isoyake | Deep | 3.14 | 1.80 | 4.62 |
| 2021-Feb | Isoyake | Deep | 3.84 | 2.40 | 5.48 |
| 2021-Mar | Isoyake | Deep | 5.01 | 3.37 | 6.86 |
| 2021-Apr | Isoyake | Deep | 6.54 | 4.46 | 8.73 |
| 2021-May | Isoyake | Deep | 7.93 | 5.47 | 10.50 |
| 2021-Jun | Isoyake | Deep | 8.58 | 6.12 | 11.27 |
| 2021-Jul | Isoyake | Deep | 8.26 | 5.91 | 10.93 |
| 2021-Aug | Isoyake | Deep | 7.02 | 4.82 | 9.26 |
| 2021-Sep | Isoyake | Deep | 5.26 | 3.56 | 7.08 |
| 2021-Oct | Isoyake | Deep | 3.57 | 2.06 | 5.09 |
| 2021-Nov | Isoyake | Deep | 2.30 | 0.97 | 3.76 |
| 2021-Dec | Isoyake | Deep | 1.47 | 0.32 | 2.73 |
| 2020-Sep | Isoyake | Shallow | 7.94 | 4.33 | 11.40 |
| 2020-Oct | Isoyake | Shallow | 6.60 | 4.40 | 8.80 |
| 2020-Nov | Isoyake | Shallow | 5.78 | 3.90 | 7.69 |
| 2020-Dec | Isoyake | Shallow | 5.52 | 3.58 | 7.66 |
| 2021-Jan | Isoyake | Shallow | 5.95 | 3.88 | 8.26 |
| 2021-Feb | Isoyake | Shallow | 7.27 | 5.25 | 9.73 |
| 2021-Mar | Isoyake | Shallow | 9.49 | 7.10 | 11.67 |
| 2021-Apr | Isoyake | Shallow | 12.39 | 9.80 | 15.16 |
| 2021-May | Isoyake | Shallow | 15.02 | 12.03 | 18.18 |
| 2021-Jun | Isoyake | Shallow | 16.27 | 13.22 | 19.36 |
| 2021-Jul | Isoyake | Shallow | 15.67 | 12.56 | 18.76 |
| 2021-Aug | Isoyake | Shallow | 13.33 | 10.37 | 16.31 |
| 2021-Sep | Isoyake | Shallow | 9.98 | 7.48 | 12.54 |
| 2021-Oct | Isoyake | Shallow | 6.78 | 4.32 | 9.31 |
| 2021-Nov | Isoyake | Shallow | 4.38 | 1.82 | 6.81 |
| 2021-Dec | Isoyake | Shallow | 2.79 | 0.64 | 5.12 |
| 2020-Sep | Vegetated | Deep | 6.27 | 3.30 | 9.10 |
| 2020-Oct | Vegetated | Deep | 5.21 | 3.28 | 7.05 |
| 2020-Nov | Vegetated | Deep | 4.56 | 2.98 | 6.18 |
| 2020-Dec | Vegetated | Deep | 4.36 | 2.77 | 6.16 |
| 2021-Jan | Vegetated | Deep | 4.70 | 2.88 | 6.54 |
| 2021-Feb | Vegetated | Deep | 5.74 | 3.76 | 7.57 |
| 2021-Mar | Vegetated | Deep | 7.49 | 5.51 | 9.55 |
| 2021-Apr | Vegetated | Deep | 9.78 | 7.36 | 12.25 |
| 2021-May | Vegetated | Deep | 11.87 | 8.99 | 14.90 |
| 2021-Jun | Vegetated | Deep | 12.86 | 9.75 | 16.06 |
| 2021-Jul | Vegetated | Deep | 12.39 | 9.08 | 15.53 |
| 2021-Aug | Vegetated | Deep | 10.54 | 7.63 | 13.70 |
| 2021-Sep | Vegetated | Deep | 7.90 | 5.32 | 10.27 |
| 2021-Oct | Vegetated | Deep | 5.37 | 3.17 | 7.60 |
| 2021-Nov | Vegetated | Deep | 3.46 | 1.41 | 5.52 |
| 2021-Dec | Vegetated | Deep | 2.21 | 0.49 | 4.07 |
| 2020-Sep | Vegetated | Shallow | 5.18 | 2.55 | 7.88 |
| 2020-Oct | Vegetated | Shallow | 4.30 | 2.57 | 6.07 |
| 2020-Nov | Vegetated | Shallow | 3.76 | 2.29 | 5.27 |
| 2020-Dec | Vegetated | Shallow | 3.59 | 2.11 | 5.11 |
| 2021-Jan | Vegetated | Shallow | 3.87 | 2.28 | 5.48 |
| 2021-Feb | Vegetated | Shallow | 4.72 | 3.04 | 6.43 |
| 2021-Mar | Vegetated | Shallow | 6.17 | 4.36 | 8.17 |
| 2021-Apr | Vegetated | Shallow | 8.06 | 5.61 | 10.53 |
| 2021-May | Vegetated | Shallow | 9.79 | 6.77 | 12.94 |
| 2021-Jun | Vegetated | Shallow | 10.62 | 6.99 | 13.90 |
| 2021-Jul | Vegetated | Shallow | 10.25 | 6.82 | 14.00 |
| 2021-Aug | Vegetated | Shallow | 8.73 | 5.35 | 11.97 |
| 2021-Sep | Vegetated | Shallow | 6.54 | 3.90 | 9.16 |
| 2021-Oct | Vegetated | Shallow | 4.44 | 2.40 | 6.66 |
| 2021-Nov | Vegetated | Shallow | 2.86 | 1.10 | 4.75 |
| 2021-Dec | Vegetated | Shallow | 1.82 | 0.37 | 3.45 |
