# Supplementary material for: The behavior of sympatric sea urchin species across an ecosystem state gradient
Source: PeerJ. 2023 Jun 13;11:e15511. doi: 10.7717/peerj.15511 (PMC10274604; doi:10.7717/peerj.15511)
Supplement: Supplemental Information 4 — The mean and 95% highest density credible interval for the expectations of the model on the monthly daily average temperature (°C) in the deep and shallow transects of the isoyake and vegetated habitat. [file peerj-11-15511-s004.docx]

| **Month** | **Habitat** | **Transect** | **Monthly daily average temperature (°C) GAM** | | |
| --- | --- | --- | --- | --- | --- |
|  |  |  | **Mean** | **Lower** | **Upper** |
| 2020-Sep | Isoyake | Deep | 24.74 | 24.15 | 25.33 |
| 2020-Oct | Isoyake | Deep | 21.85 | 21.34 | 22.39 |
| 2020-Nov | Isoyake | Deep | 19.76 | 19.29 | 20.23 |
| 2020-Dec | Isoyake | Deep | 17.28 | 16.74 | 17.78 |
| 2021-Jan | Isoyake | Deep | 14.05 | 13.51 | 14.59 |
| 2021-Feb | Isoyake | Deep | 13.24 | 12.75 | 13.73 |
| 2021-Mar | Isoyake | Deep | 15.06 | 14.60 | 15.55 |
| 2021-Apr | Isoyake | Deep | 17.53 | 17.01 | 18.07 |
| 2021-May | Isoyake | Deep | 19.19 | 18.65 | 19.72 |
| 2021-Jun | Isoyake | Deep | 21.73 | 21.22 | 22.23 |
| 2021-Jul | Isoyake | Deep | 24.83 | 24.30 | 25.42 |
| 2021-Aug | Isoyake | Deep | 26.79 | 26.20 | 27.36 |
| 2021-Sep | Isoyake | Deep | 26.17 | 25.61 | 26.75 |
| 2021-Oct | Isoyake | Deep | 23.62 | 23.10 | 24.13 |
| 2021-Nov | Isoyake | Deep | 20.18 | 19.56 | 20.80 |
| 2021-Dec | Isoyake | Deep | 16.56 | 15.76 | 17.33 |
| 2020-Sep | Isoyake | Shallow | 25.00 | 24.41 | 25.61 |
| 2020-Oct | Isoyake | Shallow | 22.12 | 21.58 | 22.63 |
| 2020-Nov | Isoyake | Shallow | 20.03 | 19.54 | 20.49 |
| 2020-Dec | Isoyake | Shallow | 17.54 | 17.02 | 18.05 |
| 2021-Jan | Isoyake | Shallow | 14.32 | 13.80 | 14.88 |
| 2021-Feb | Isoyake | Shallow | 13.51 | 13.01 | 14.01 |
| 2021-Mar | Isoyake | Shallow | 15.33 | 14.86 | 15.83 |
| 2021-Apr | Isoyake | Shallow | 17.80 | 17.26 | 18.35 |
| 2021-May | Isoyake | Shallow | 19.46 | 18.93 | 20.01 |
| 2021-Jun | Isoyake | Shallow | 21.99 | 21.49 | 22.51 |
| 2021-Jul | Isoyake | Shallow | 25.10 | 24.55 | 25.67 |
| 2021-Aug | Isoyake | Shallow | 27.05 | 26.50 | 27.68 |
| 2021-Sep | Isoyake | Shallow | 26.43 | 25.88 | 27.06 |
| 2021-Oct | Isoyake | Shallow | 23.88 | 23.36 | 24.40 |
| 2021-Nov | Isoyake | Shallow | 20.45 | 19.83 | 21.08 |
| 2021-Dec | Isoyake | Shallow | 16.83 | 16.06 | 17.61 |
| 2020-Sep | Vegetated | Deep | 25.01 | 24.39 | 25.58 |
| 2020-Oct | Vegetated | Deep | 22.12 | 21.59 | 22.65 |
| 2020-Nov | Vegetated | Deep | 20.03 | 19.54 | 20.50 |
| 2020-Dec | Vegetated | Deep | 17.54 | 17.01 | 18.06 |
| 2021-Jan | Vegetated | Deep | 14.32 | 13.77 | 14.87 |
| 2021-Feb | Vegetated | Deep | 13.51 | 13.00 | 14.01 |
| 2021-Mar | Vegetated | Deep | 15.33 | 14.82 | 15.80 |
| 2021-Apr | Vegetated | Deep | 17.80 | 17.24 | 18.33 |
| 2021-May | Vegetated | Deep | 19.46 | 18.91 | 19.97 |
| 2021-Jun | Vegetated | Deep | 22.00 | 21.50 | 22.55 |
| 2021-Jul | Vegetated | Deep | 25.10 | 24.50 | 25.70 |
| 2021-Aug | Vegetated | Deep | 27.05 | 26.44 | 27.65 |
| 2021-Sep | Vegetated | Deep | 26.43 | 25.88 | 27.05 |
| 2021-Oct | Vegetated | Deep | 23.89 | 23.35 | 24.42 |
| 2021-Nov | Vegetated | Deep | 20.45 | 19.79 | 21.16 |
| 2021-Dec | Vegetated | Deep | 16.83 | 15.99 | 17.69 |
| 2020-Sep | Vegetated | Shallow | 25.14 | 24.51 | 25.74 |
| 2020-Oct | Vegetated | Shallow | 22.25 | 21.69 | 22.78 |
| 2020-Nov | Vegetated | Shallow | 20.16 | 19.66 | 20.68 |
| 2020-Dec | Vegetated | Shallow | 17.68 | 17.10 | 18.20 |
| 2021-Jan | Vegetated | Shallow | 14.46 | 13.91 | 15.04 |
| 2021-Feb | Vegetated | Shallow | 13.65 | 13.12 | 14.16 |
| 2021-Mar | Vegetated | Shallow | 15.46 | 14.94 | 15.95 |
| 2021-Apr | Vegetated | Shallow | 17.94 | 17.39 | 18.52 |
| 2021-May | Vegetated | Shallow | 19.60 | 19.05 | 20.17 |
| 2021-Jun | Vegetated | Shallow | 22.13 | 21.57 | 22.67 |
| 2021-Jul | Vegetated | Shallow | 25.23 | 24.62 | 25.88 |
| 2021-Aug | Vegetated | Shallow | 27.19 | 26.52 | 27.86 |
| 2021-Sep | Vegetated | Shallow | 26.57 | 25.89 | 27.22 |
| 2021-Oct | Vegetated | Shallow | 24.02 | 23.38 | 24.62 |
| 2021-Nov | Vegetated | Shallow | 20.59 | 19.85 | 21.28 |
| 2021-Dec | Vegetated | Shallow | 16.96 | 16.07 | 17.80 |
