# Supplementary material for: The behavior of sympatric sea urchin species across an ecosystem state gradient
Source: PeerJ. 2023 Jun 13;11:e15511. doi: 10.7717/peerj.15511 (PMC10274604; doi:10.7717/peerj.15511)
Supplement: Supplemental Information 5 — The mean and 95% highest density credible interval for the expectations of the model on benthic rugosity in the deep and shallow transects of the isoyake and vegetated habitat. [file peerj-11-15511-s005.docx]

| **Habitat** | **Transect** | **Rugosity GLM** | | |
| --- | --- | --- | --- | --- |
|  |  | **Mean** | **Lower** | **Upper** |
| Isoyake | deep | 1.16 | 1.12 | 1.2 |
| Isoyake | shallow | 1.23 | 1.17 | 1.28 |
| Vegetated | deep | 1.38 | 1.33 | 1.44 |
| Vegetated | shallow | 1.37 | 1.32 | 1.41 |
