# Supplementary material for: The behavior of sympatric sea urchin species across an ecosystem state gradient
Source: PeerJ. 2023 Jun 13;11:e15511. doi: 10.7717/peerj.15511 (PMC10274604; doi:10.7717/peerj.15511)
Supplement: Supplemental Information 6 — The mean and 95% highest density credible interval for the expectations of the model on the percent benthic cover in the deep and shallow transects of the isoyake and vegetated habitat. [file peerj-11-15511-s006.docx]

| **Month** | **Habitat** | **Transect** | **Element** | **Benthic cover (%) GAM** | | |
| --- | --- | --- | --- | --- | --- | --- |
|  |  |  |  | **Mean** | **Lower** | **Upper** |
| 2020-Sep | Isoyake | Deep | Coralline | 1.54 | 0.08 | 3.45 |
| 2020-Oct | Isoyake | Deep | Coralline | 1.67 | 0.30 | 3.22 |
| 2020-Nov | Isoyake | Deep | Coralline | 1.80 | 0.46 | 3.32 |
| 2020-Dec | Isoyake | Deep | Coralline | 1.97 | 0.47 | 3.71 |
| 2021-Jan | Isoyake | Deep | Coralline | 2.20 | 0.54 | 4.04 |
| 2021-Feb | Isoyake | Deep | Coralline | 2.44 | 0.78 | 4.26 |
| 2021-Mar | Isoyake | Deep | Coralline | 2.61 | 0.98 | 4.45 |
| 2021-Apr | Isoyake | Deep | Coralline | 2.76 | 1.09 | 4.65 |
| 2021-May | Isoyake | Deep | Coralline | 2.88 | 1.20 | 4.89 |
| 2021-Jun | Isoyake | Deep | Coralline | 2.95 | 1.22 | 5.03 |
| 2021-Jul | Isoyake | Deep | Coralline | 2.86 | 0.86 | 5.13 |
| 2021-Aug | Isoyake | Deep | Coralline | 2.45 | 0.10 | 5.02 |
| 2021-Sep | Isoyake | Deep | Coralline | 2.17 | 0.00 | 4.84 |
| 2021-Oct | Isoyake | Deep | Coralline | 2.18 | 0.01 | 4.70 |
| 2021-Nov | Isoyake | Deep | Coralline | 2.48 | 0.29 | 5.22 |
| 2021-Dec | Isoyake | Deep | Coralline | 2.83 | 0.05 | 6.29 |
| 2020-Sep | Isoyake | Deep | Macroalgae | 0.15 | 0.00 | 0.53 |
| 2020-Oct | Isoyake | Deep | Macroalgae | 0.25 | 0.00 | 1.18 |
| 2020-Nov | Isoyake | Deep | Macroalgae | 0.78 | 0.00 | 3.67 |
| 2020-Dec | Isoyake | Deep | Macroalgae | 3.74 | 0.00 | 13.25 |
| 2021-Jan | Isoyake | Deep | Macroalgae | 9.46 | 0.00 | 23.03 |
| 2021-Feb | Isoyake | Deep | Macroalgae | 11.62 | 1.85 | 22.99 |
| 2021-Mar | Isoyake | Deep | Macroalgae | 10.46 | 3.27 | 19.04 |
| 2021-Apr | Isoyake | Deep | Macroalgae | 8.34 | 2.70 | 14.88 |
| 2021-May | Isoyake | Deep | Macroalgae | 6.31 | 1.80 | 11.50 |
| 2021-Jun | Isoyake | Deep | Macroalgae | 4.67 | 1.08 | 8.75 |
| 2021-Jul | Isoyake | Deep | Macroalgae | 3.56 | 0.59 | 7.47 |
| 2021-Aug | Isoyake | Deep | Macroalgae | 2.61 | 0.03 | 6.18 |
| 2021-Sep | Isoyake | Deep | Macroalgae | 1.10 | 0.00 | 3.67 |
| 2021-Oct | Isoyake | Deep | Macroalgae | 0.27 | 0.00 | 1.19 |
| 2021-Nov | Isoyake | Deep | Macroalgae | 0.12 | 0.00 | 0.52 |
| 2021-Dec | Isoyake | Deep | Macroalgae | 0.10 | 0.00 | 0.27 |
| 2020-Sep | Isoyake | Deep | Substrate | 97.05 | 93.52 | 99.80 |
| 2020-Oct | Isoyake | Deep | Substrate | 96.52 | 93.11 | 99.37 |
| 2020-Nov | Isoyake | Deep | Substrate | 95.64 | 92.21 | 98.66 |
| 2020-Dec | Isoyake | Deep | Substrate | 94.34 | 90.36 | 97.73 |
| 2021-Jan | Isoyake | Deep | Substrate | 92.72 | 87.86 | 97.20 |
| 2021-Feb | Isoyake | Deep | Substrate | 91.17 | 85.38 | 96.59 |
| 2021-Mar | Isoyake | Deep | Substrate | 90.10 | 83.57 | 95.92 |
| 2021-Apr | Isoyake | Deep | Substrate | 89.95 | 83.36 | 95.87 |
| 2021-May | Isoyake | Deep | Substrate | 90.95 | 85.25 | 95.94 |
| 2021-Jun | Isoyake | Deep | Substrate | 92.75 | 88.51 | 96.50 |
| 2021-Jul | Isoyake | Deep | Substrate | 94.31 | 90.44 | 97.91 |
| 2021-Aug | Isoyake | Deep | Substrate | 95.29 | 91.28 | 98.70 |
| 2021-Sep | Isoyake | Deep | Substrate | 95.78 | 91.75 | 99.02 |
| 2021-Oct | Isoyake | Deep | Substrate | 95.93 | 91.93 | 99.10 |
| 2021-Nov | Isoyake | Deep | Substrate | 95.79 | 91.56 | 99.14 |
| 2021-Dec | Isoyake | Deep | Substrate | 95.32 | 90.38 | 99.33 |
| 2020-Sep | Isoyake | Deep | Turf | 0.07 | 0.00 | 0.30 |
| 2020-Oct | Isoyake | Deep | Turf | 0.13 | 0.00 | 0.50 |
| 2020-Nov | Isoyake | Deep | Turf | 0.27 | 0.00 | 0.93 |
| 2020-Dec | Isoyake | Deep | Turf | 0.57 | 0.01 | 1.78 |
| 2021-Jan | Isoyake | Deep | Turf | 1.01 | 0.03 | 2.92 |
| 2021-Feb | Isoyake | Deep | Turf | 1.53 | 0.12 | 3.90 |
| 2021-Mar | Isoyake | Deep | Turf | 2.05 | 0.24 | 4.66 |
| 2021-Apr | Isoyake | Deep | Turf | 2.40 | 0.41 | 5.26 |
| 2021-May | Isoyake | Deep | Turf | 2.30 | 0.31 | 5.26 |
| 2021-Jun | Isoyake | Deep | Turf | 1.68 | 0.14 | 4.21 |
| 2021-Jul | Isoyake | Deep | Turf | 0.98 | 0.03 | 2.76 |
| 2021-Aug | Isoyake | Deep | Turf | 0.51 | 0.00 | 1.66 |
| 2021-Sep | Isoyake | Deep | Turf | 0.25 | 0.00 | 0.92 |
| 2021-Oct | Isoyake | Deep | Turf | 0.14 | 0.00 | 0.54 |
| 2021-Nov | Isoyake | Deep | Turf | 0.09 | 0.00 | 0.40 |
| 2021-Dec | Isoyake | Deep | Turf | 0.09 | 0.00 | 0.38 |
| 2020-Sep | Isoyake | Shallow | Coralline | 5.40 | 1.46 | 9.81 |
| 2020-Oct | Isoyake | Shallow | Coralline | 5.66 | 2.10 | 9.18 |
| 2020-Nov | Isoyake | Shallow | Coralline | 5.94 | 2.81 | 9.49 |
| 2020-Dec | Isoyake | Shallow | Coralline | 6.19 | 3.00 | 10.05 |
| 2021-Jan | Isoyake | Shallow | Coralline | 6.38 | 2.96 | 10.32 |
| 2021-Feb | Isoyake | Shallow | Coralline | 6.49 | 3.34 | 10.33 |
| 2021-Mar | Isoyake | Shallow | Coralline | 6.54 | 3.39 | 9.96 |
| 2021-Apr | Isoyake | Shallow | Coralline | 6.52 | 3.46 | 10.13 |
| 2021-May | Isoyake | Shallow | Coralline | 6.45 | 3.17 | 9.96 |
| 2021-Jun | Isoyake | Shallow | Coralline | 6.30 | 3.07 | 9.48 |
| 2021-Jul | Isoyake | Shallow | Coralline | 6.14 | 3.20 | 9.31 |
| 2021-Aug | Isoyake | Shallow | Coralline | 6.00 | 3.09 | 9.23 |
| 2021-Sep | Isoyake | Shallow | Coralline | 5.92 | 2.97 | 9.04 |
| 2021-Oct | Isoyake | Shallow | Coralline | 5.89 | 3.00 | 9.26 |
| 2021-Nov | Isoyake | Shallow | Coralline | 5.92 | 2.22 | 9.61 |
| 2021-Dec | Isoyake | Shallow | Coralline | 6.00 | 1.65 | 11.42 |
| 2020-Sep | Isoyake | Shallow | Macroalgae | 0.12 | 0.00 | 0.28 |
| 2020-Oct | Isoyake | Shallow | Macroalgae | 0.26 | 0.00 | 1.12 |
| 2020-Nov | Isoyake | Shallow | Macroalgae | 1.14 | 0.00 | 5.41 |
| 2020-Dec | Isoyake | Shallow | Macroalgae | 7.35 | 0.00 | 24.42 |
| 2021-Jan | Isoyake | Shallow | Macroalgae | 23.69 | 0.00 | 48.97 |
| 2021-Feb | Isoyake | Shallow | Macroalgae | 32.10 | 10.41 | 53.75 |
| 2021-Mar | Isoyake | Shallow | Macroalgae | 27.41 | 12.04 | 41.51 |
| 2021-Apr | Isoyake | Shallow | Macroalgae | 17.73 | 8.09 | 28.29 |
| 2021-May | Isoyake | Shallow | Macroalgae | 9.70 | 3.63 | 16.70 |
| 2021-Jun | Isoyake | Shallow | Macroalgae | 5.07 | 1.36 | 9.78 |
| 2021-Jul | Isoyake | Shallow | Macroalgae | 2.92 | 0.31 | 6.63 |
| 2021-Aug | Isoyake | Shallow | Macroalgae | 1.82 | 0.03 | 5.04 |
| 2021-Sep | Isoyake | Shallow | Macroalgae | 0.77 | 0.00 | 2.80 |
| 2021-Oct | Isoyake | Shallow | Macroalgae | 0.23 | 0.00 | 1.07 |
| 2021-Nov | Isoyake | Shallow | Macroalgae | 0.14 | 0.00 | 0.59 |
| 2021-Dec | Isoyake | Shallow | Macroalgae | 0.14 | 0.00 | 0.39 |
| 2020-Sep | Isoyake | Shallow | Substrate | 95.82 | 91.56 | 99.37 |
| 2020-Oct | Isoyake | Shallow | Substrate | 92.81 | 87.84 | 97.57 |
| 2020-Nov | Isoyake | Shallow | Substrate | 86.99 | 80.73 | 93.01 |
| 2020-Dec | Isoyake | Shallow | Substrate | 78.38 | 69.50 | 87.26 |
| 2021-Jan | Isoyake | Shallow | Substrate | 69.91 | 58.46 | 82.02 |
| 2021-Feb | Isoyake | Shallow | Substrate | 65.23 | 53.77 | 77.02 |
| 2021-Mar | Isoyake | Shallow | Substrate | 65.33 | 55.04 | 75.80 |
| 2021-Apr | Isoyake | Shallow | Substrate | 69.67 | 60.01 | 79.47 |
| 2021-May | Isoyake | Shallow | Substrate | 76.88 | 67.86 | 85.22 |
| 2021-Jun | Isoyake | Shallow | Substrate | 84.57 | 77.93 | 90.73 |
| 2021-Jul | Isoyake | Shallow | Substrate | 90.02 | 84.50 | 95.00 |
| 2021-Aug | Isoyake | Shallow | Substrate | 92.92 | 87.92 | 97.20 |
| 2021-Sep | Isoyake | Shallow | Substrate | 94.05 | 89.66 | 97.97 |
| 2021-Oct | Isoyake | Shallow | Substrate | 94.06 | 89.77 | 97.59 |
| 2021-Nov | Isoyake | Shallow | Substrate | 93.15 | 88.30 | 97.59 |
| 2021-Dec | Isoyake | Shallow | Substrate | 91.15 | 83.36 | 98.10 |
| 2020-Sep | Isoyake | Shallow | Turf | 0.09 | 0.00 | 0.40 |
| 2020-Oct | Isoyake | Shallow | Turf | 0.17 | 0.00 | 0.65 |
| 2020-Nov | Isoyake | Shallow | Turf | 0.35 | 0.00 | 1.23 |
| 2020-Dec | Isoyake | Shallow | Turf | 0.72 | 0.01 | 2.33 |
| 2021-Jan | Isoyake | Shallow | Turf | 1.27 | 0.03 | 3.70 |
| 2021-Feb | Isoyake | Shallow | Turf | 1.90 | 0.12 | 4.83 |
| 2021-Mar | Isoyake | Shallow | Turf | 2.52 | 0.23 | 5.66 |
| 2021-Apr | Isoyake | Shallow | Turf | 2.94 | 0.46 | 6.44 |
| 2021-May | Isoyake | Shallow | Turf | 2.81 | 0.41 | 6.34 |
| 2021-Jun | Isoyake | Shallow | Turf | 2.06 | 0.20 | 4.99 |
| 2021-Jul | Isoyake | Shallow | Turf | 1.21 | 0.02 | 3.33 |
| 2021-Aug | Isoyake | Shallow | Turf | 0.63 | 0.00 | 2.04 |
| 2021-Sep | Isoyake | Shallow | Turf | 0.32 | 0.00 | 1.14 |
| 2021-Oct | Isoyake | Shallow | Turf | 0.17 | 0.00 | 0.67 |
| 2021-Nov | Isoyake | Shallow | Turf | 0.12 | 0.00 | 0.49 |
| 2021-Dec | Isoyake | Shallow | Turf | 0.11 | 0.00 | 0.47 |
| 2020-Sep | Vegetated | Deep | Coralline | 4.71 | 1.28 | 8.77 |
| 2020-Oct | Vegetated | Deep | Coralline | 5.20 | 2.00 | 8.60 |
| 2020-Nov | Vegetated | Deep | Coralline | 5.74 | 2.61 | 9.06 |
| 2020-Dec | Vegetated | Deep | Coralline | 6.27 | 2.97 | 9.91 |
| 2021-Jan | Vegetated | Deep | Coralline | 6.73 | 3.32 | 10.75 |
| 2021-Feb | Vegetated | Deep | Coralline | 7.09 | 3.79 | 11.02 |
| 2021-Mar | Vegetated | Deep | Coralline | 7.44 | 4.27 | 11.27 |
| 2021-Apr | Vegetated | Deep | Coralline | 7.87 | 4.18 | 11.61 |
| 2021-May | Vegetated | Deep | Coralline | 8.50 | 4.55 | 12.58 |
| 2021-Jun | Vegetated | Deep | Coralline | 9.42 | 5.31 | 13.51 |
| 2021-Jul | Vegetated | Deep | Coralline | 10.58 | 6.53 | 14.95 |
| 2021-Aug | Vegetated | Deep | Coralline | 11.89 | 7.11 | 16.82 |
| 2021-Sep | Vegetated | Deep | Coralline | 13.13 | 7.83 | 18.71 |
| 2021-Oct | Vegetated | Deep | Coralline | 14.17 | 8.73 | 20.18 |
| 2021-Nov | Vegetated | Deep | Coralline | 15.00 | 8.60 | 22.15 |
| 2021-Dec | Vegetated | Deep | Coralline | 15.69 | 6.52 | 24.97 |
| 2020-Sep | Vegetated | Deep | Macroalgae | 0.17 | 0.00 | 0.69 |
| 2020-Oct | Vegetated | Deep | Macroalgae | 0.31 | 0.00 | 1.49 |
| 2020-Nov | Vegetated | Deep | Macroalgae | 0.94 | 0.00 | 4.04 |
| 2020-Dec | Vegetated | Deep | Macroalgae | 3.59 | 0.00 | 11.44 |
| 2021-Jan | Vegetated | Deep | Macroalgae | 7.91 | 0.00 | 18.48 |
| 2021-Feb | Vegetated | Deep | Macroalgae | 11.33 | 2.42 | 20.97 |
| 2021-Mar | Vegetated | Deep | Macroalgae | 13.18 | 4.81 | 22.37 |
| 2021-Apr | Vegetated | Deep | Macroalgae | 13.44 | 4.82 | 22.81 |
| 2021-May | Vegetated | Deep | Macroalgae | 11.89 | 4.52 | 20.49 |
| 2021-Jun | Vegetated | Deep | Macroalgae | 8.99 | 2.90 | 15.82 |
| 2021-Jul | Vegetated | Deep | Macroalgae | 5.98 | 0.53 | 12.45 |
| 2021-Aug | Vegetated | Deep | Macroalgae | 2.78 | 0.00 | 8.03 |
| 2021-Sep | Vegetated | Deep | Macroalgae | 0.89 | 0.00 | 3.58 |
| 2021-Oct | Vegetated | Deep | Macroalgae | 0.32 | 0.00 | 1.42 |
| 2021-Nov | Vegetated | Deep | Macroalgae | 0.19 | 0.00 | 0.85 |
| 2021-Dec | Vegetated | Deep | Macroalgae | 0.20 | 0.00 | 0.78 |
| 2020-Sep | Vegetated | Deep | Substrate | 93.76 | 88.35 | 98.38 |
| 2020-Oct | Vegetated | Deep | Substrate | 92.32 | 87.32 | 96.97 |
| 2020-Nov | Vegetated | Deep | Substrate | 90.36 | 85.34 | 95.07 |
| 2020-Dec | Vegetated | Deep | Substrate | 87.96 | 81.89 | 93.52 |
| 2021-Jan | Vegetated | Deep | Substrate | 85.49 | 77.90 | 92.08 |
| 2021-Feb | Vegetated | Deep | Substrate | 83.41 | 75.37 | 90.51 |
| 2021-Mar | Vegetated | Deep | Substrate | 81.95 | 74.19 | 89.44 |
| 2021-Apr | Vegetated | Deep | Substrate | 81.17 | 72.84 | 88.26 |
| 2021-May | Vegetated | Deep | Substrate | 81.16 | 73.46 | 88.24 |
| 2021-Jun | Vegetated | Deep | Substrate | 81.82 | 74.99 | 88.03 |
| 2021-Jul | Vegetated | Deep | Substrate | 82.74 | 76.55 | 88.70 |
| 2021-Aug | Vegetated | Deep | Substrate | 83.53 | 76.54 | 89.57 |
| 2021-Sep | Vegetated | Deep | Substrate | 83.98 | 76.73 | 90.19 |
| 2021-Oct | Vegetated | Deep | Substrate | 84.03 | 77.24 | 90.70 |
| 2021-Nov | Vegetated | Deep | Substrate | 83.72 | 76.31 | 91.28 |
| 2021-Dec | Vegetated | Deep | Substrate | 83.06 | 72.63 | 92.54 |
| 2020-Sep | Vegetated | Deep | Turf | 0.09 | 0.00 | 0.38 |
| 2020-Oct | Vegetated | Deep | Turf | 0.16 | 0.00 | 0.62 |
| 2020-Nov | Vegetated | Deep | Turf | 0.33 | 0.00 | 1.20 |
| 2020-Dec | Vegetated | Deep | Turf | 0.69 | 0.01 | 2.22 |
| 2021-Jan | Vegetated | Deep | Turf | 1.21 | 0.03 | 3.48 |
| 2021-Feb | Vegetated | Deep | Turf | 1.81 | 0.10 | 4.63 |
| 2021-Mar | Vegetated | Deep | Turf | 2.40 | 0.33 | 5.59 |
| 2021-Apr | Vegetated | Deep | Turf | 2.81 | 0.36 | 6.22 |
| 2021-May | Vegetated | Deep | Turf | 2.70 | 0.30 | 6.30 |
| 2021-Jun | Vegetated | Deep | Turf | 1.99 | 0.18 | 5.03 |
| 2021-Jul | Vegetated | Deep | Turf | 1.17 | 0.05 | 3.39 |
| 2021-Aug | Vegetated | Deep | Turf | 0.61 | 0.00 | 2.00 |
| 2021-Sep | Vegetated | Deep | Turf | 0.31 | 0.00 | 1.13 |
| 2021-Oct | Vegetated | Deep | Turf | 0.17 | 0.00 | 0.67 |
| 2021-Nov | Vegetated | Deep | Turf | 0.12 | 0.00 | 0.50 |
| 2021-Dec | Vegetated | Deep | Turf | 0.11 | 0.00 | 0.48 |
| 2020-Sep | Vegetated | Shallow | Coralline | 36.15 | 20.30 | 52.93 |
| 2020-Oct | Vegetated | Shallow | Coralline | 53.60 | 41.21 | 66.21 |
| 2020-Nov | Vegetated | Shallow | Coralline | 66.90 | 54.55 | 77.83 |
| 2020-Dec | Vegetated | Shallow | Coralline | 72.93 | 59.94 | 84.82 |
| 2021-Jan | Vegetated | Shallow | Coralline | 72.15 | 57.83 | 84.34 |
| 2021-Feb | Vegetated | Shallow | Coralline | 63.41 | 49.84 | 75.97 |
| 2021-Mar | Vegetated | Shallow | Coralline | 47.60 | 36.45 | 58.87 |
| 2021-Apr | Vegetated | Shallow | Coralline | 32.37 | 21.97 | 42.06 |
| 2021-May | Vegetated | Shallow | Coralline | 25.38 | 16.13 | 34.91 |
| 2021-Jun | Vegetated | Shallow | Coralline | 28.34 | 19.66 | 37.57 |
| 2021-Jul | Vegetated | Shallow | Coralline | 39.16 | 29.67 | 49.33 |
| 2021-Aug | Vegetated | Shallow | Coralline | 53.19 | 41.49 | 65.70 |
| 2021-Sep | Vegetated | Shallow | Coralline | 63.36 | 50.95 | 75.15 |
| 2021-Oct | Vegetated | Shallow | Coralline | 68.07 | 56.79 | 78.84 |
| 2021-Nov | Vegetated | Shallow | Coralline | 69.09 | 55.72 | 81.04 |
| 2021-Dec | Vegetated | Shallow | Coralline | 67.65 | 46.46 | 84.56 |
| 2020-Sep | Vegetated | Shallow | Macroalgae | 0.06 | 0.00 | 0.14 |
| 2020-Oct | Vegetated | Shallow | Macroalgae | 0.07 | 0.00 | 0.29 |
| 2020-Nov | Vegetated | Shallow | Macroalgae | 0.18 | 0.00 | 0.86 |
| 2020-Dec | Vegetated | Shallow | Macroalgae | 0.89 | 0.00 | 3.81 |
| 2021-Jan | Vegetated | Shallow | Macroalgae | 3.64 | 0.00 | 11.51 |
| 2021-Feb | Vegetated | Shallow | Macroalgae | 9.21 | 0.74 | 19.39 |
| 2021-Mar | Vegetated | Shallow | Macroalgae | 18.03 | 7.54 | 29.23 |
| 2021-Apr | Vegetated | Shallow | Macroalgae | 28.35 | 14.63 | 41.34 |
| 2021-May | Vegetated | Shallow | Macroalgae | 33.36 | 16.60 | 49.87 |
| 2021-Jun | Vegetated | Shallow | Macroalgae | 28.15 | 14.63 | 42.62 |
| 2021-Jul | Vegetated | Shallow | Macroalgae | 17.68 | 8.14 | 27.70 |
| 2021-Aug | Vegetated | Shallow | Macroalgae | 8.98 | 2.64 | 16.96 |
| 2021-Sep | Vegetated | Shallow | Macroalgae | 3.52 | 0.06 | 8.71 |
| 2021-Oct | Vegetated | Shallow | Macroalgae | 0.42 | 0.00 | 1.82 |
| 2021-Nov | Vegetated | Shallow | Macroalgae | 0.07 | 0.00 | 0.29 |
| 2021-Dec | Vegetated | Shallow | Macroalgae | 0.03 | 0.00 | 0.05 |
| 2020-Sep | Vegetated | Shallow | Substrate | 58.65 | 42.46 | 74.00 |
| 2020-Oct | Vegetated | Shallow | Substrate | 44.23 | 34.01 | 56.03 |
| 2020-Nov | Vegetated | Shallow | Substrate | 32.12 | 22.92 | 42.32 |
| 2020-Dec | Vegetated | Shallow | Substrate | 24.19 | 14.60 | 33.77 |
| 2021-Jan | Vegetated | Shallow | Substrate | 19.82 | 10.99 | 28.82 |
| 2021-Feb | Vegetated | Shallow | Substrate | 17.91 | 10.00 | 26.08 |
| 2021-Mar | Vegetated | Shallow | Substrate | 17.63 | 10.46 | 25.43 |
| 2021-Apr | Vegetated | Shallow | Substrate | 18.62 | 11.54 | 26.60 |
| 2021-May | Vegetated | Shallow | Substrate | 20.70 | 12.75 | 28.62 |
| 2021-Jun | Vegetated | Shallow | Substrate | 23.71 | 15.86 | 32.01 |
| 2021-Jul | Vegetated | Shallow | Substrate | 27.34 | 18.21 | 35.38 |
| 2021-Aug | Vegetated | Shallow | Substrate | 30.81 | 21.28 | 40.61 |
| 2021-Sep | Vegetated | Shallow | Substrate | 32.94 | 22.76 | 42.81 |
| 2021-Oct | Vegetated | Shallow | Substrate | 33.32 | 24.22 | 42.63 |
| 2021-Nov | Vegetated | Shallow | Substrate | 32.62 | 22.59 | 42.82 |
| 2021-Dec | Vegetated | Shallow | Substrate | 31.63 | 18.58 | 45.97 |
| 2020-Sep | Vegetated | Shallow | Turf | 0.92 | 0.00 | 3.63 |
| 2020-Oct | Vegetated | Shallow | Turf | 1.61 | 0.00 | 5.08 |
| 2020-Nov | Vegetated | Shallow | Turf | 3.01 | 0.07 | 7.29 |
| 2020-Dec | Vegetated | Shallow | Turf | 5.08 | 0.80 | 10.62 |
| 2021-Jan | Vegetated | Shallow | Turf | 7.42 | 2.13 | 14.04 |
| 2021-Feb | Vegetated | Shallow | Turf | 10.04 | 3.71 | 16.87 |
| 2021-Mar | Vegetated | Shallow | Turf | 12.88 | 6.12 | 21.09 |
| 2021-Apr | Vegetated | Shallow | Turf | 15.16 | 6.44 | 24.49 |
| 2021-May | Vegetated | Shallow | Turf | 15.39 | 6.48 | 25.70 |
| 2021-Jun | Vegetated | Shallow | Turf | 12.74 | 5.08 | 20.52 |
| 2021-Jul | Vegetated | Shallow | Turf | 8.74 | 2.63 | 15.14 |
| 2021-Aug | Vegetated | Shallow | Turf | 5.23 | 0.78 | 10.80 |
| 2021-Sep | Vegetated | Shallow | Turf | 2.96 | 0.13 | 7.25 |
| 2021-Oct | Vegetated | Shallow | Turf | 1.73 | 0.01 | 5.15 |
| 2021-Nov | Vegetated | Shallow | Turf | 1.19 | 0.00 | 4.46 |
| 2021-Dec | Vegetated | Shallow | Turf | 1.01 | 0.00 | 4.45 |
