# Supplementary material for: The behavior of sympatric sea urchin species across an ecosystem state gradient
Source: PeerJ. 2023 Jun 13;11:e15511. doi: 10.7717/peerj.15511 (PMC10274604; doi:10.7717/peerj.15511)
Supplement: Supplemental Information 7 — The relative importance is the ratio of the absolute values of the coefficients for the model’s main effects excluding the covariate interactions and effects of the time smoother, in the case of Generalized Additive Models (GAM). [file peerj-11-15511-s007.docx]

| **Observation** | **Parameter** | **Relative importance** | **Model distribution** |
| --- | --- | --- | --- |
| Wave height | IH wave height | 0.600 | Gamma |
|  | VH wave height | 0.400 |  |
| Light | IH deep | 0.222 | Gaussian |
|  | IH shallow | 0.259 |  |
|  | VH deep | 0.241 |  |
|  | VH shallow | 0.278 |  |
| Temperature | IH deep | 0.248 | Gaussian |
|  | IH shallow | 0.250 |  |
|  | VH deep | 0.250 |  |
|  | VH shallow | 0.252 |  |
| Rugosity | IH deep | 0.138 | Gaussian |
|  | IH shallow | 0.194 |  |
|  | VH deep | 0.306 |  |
|  | VH shallow | 0.362 |  |
| Coralline cover | IH deep | 0.383 | Zero-inflated Beta |
|  | IH shallow | 0.261 |  |
|  | VH deep | 0.239 |  |
|  | VH shallow | 0.117 |  |
| Macroalgae cover | IH deep | 0.250 | Zero-inflated Beta |
|  | IH shallow | 0.251 |  |
|  | VH deep | 0.249 |  |
|  | VH shallow | 0.250 |  |
| Turf algae cover | IH deep | 0.283 | Zero-inflated Beta |
|  | IH shallow | 0.249 |  |
|  | VH deep | 0.251 |  |
|  | VH shallow | 0.217 |  |
| Substrate cover | IH deep | 0.446 | Zero-one inflated Beta |
|  | IH shallow | 0.258 |  |
|  | VH deep | 0.242 |  |
|  | VH shallow | 0.054 |  |
| Microhabitat | IH deep: Small *D. savignyi* in crevices | 0.020 | Beta |
|  | IH deep: Medium *D. savignyi* in crevices | 0.021 |  |
|  | IH deep: Small *D. savignyi* as free-living | 0.017 |  |
|  | IH deep: Medium *D. savignyi* as free-living | 0.018 |  |
|  | IH deep: Small *D. setosum* in crevices | 0.014 |  |
|  | IH deep: Medium *D. setosum* in crevices | 0.015 |  |
|  | IH deep: Medium *D. setosum* as free-living | 0.012 |  |
|  | IH deep: Large *D. setosum* as free-living | 0.015 |  |
|  | IH deep: Medium *H. crassispina* in crevices | 0.022 |  |
|  | IH deep: Medium *H. crassispina* as free-living | 0.019 |  |
|  | IH shallow: Small *D. savignyi* in pits | 0.022 |  |
|  | IH shallow: Medium *D. savignyi* in pits | 0.023 |  |
|  | IH shallow: Small *D. savignyi* in crevices | 0.022 |  |
|  | IH shallow: Medium *D. savignyi* in crevices | 0.023 |  |
|  | IH shallow: Small *D. savignyi* as free-living | 0.019 |  |
|  | IH shallow: Medium *D. savignyi* as free-living | 0.020 |  |
|  | IH shallow: Large *D. savignyi* as free-living | 0.023 |  |
|  | IH shallow: Small *D. setosum* in pits | 0.016 |  |
|  | IH shallow: Medium *D. setosum* in pits | 0.017 |  |
|  | IH shallow: Small *D. setosum* in crevices | 0.016 |  |
|  | IH shallow: Medium *D. setosum* in crevices | 0.017 |  |
|  | IH shallow: Small *D. setosum* as free-living | 0.013 |  |
|  | IH shallow: Medium *D. setosum* as free-living | 0.014 |  |
|  | IH shallow: Medium *H. crassispina* in pits | 0.024 |  |
|  | IH shallow: Medium *H. crassispina* in crevices | 0.024 |  |
|  | IH shallow: Medium *H. crassispina* as free-living | 0.021 |  |
|  | VH deep: Small *D. savignyi* in crevices | 0.019 |  |
|  | VH deep: Large *D. savignyi* in crevices | 0.023 |  |
|  | VH deep: Small *D. savignyi* as free-living | 0.016 |  |
|  | VH deep: Large *D. savignyi* as free-living | 0.020 |  |
|  | VH deep: Small *D. setosum* in crevices | 0.013 |  |
|  | VH deep: Large *D. setosum* in crevices | 0.017 |  |
|  | VH deep: Small *D. setosum* as free-living | 0.010 |  |
|  | VH deep: Large *D. setosum* as free-living | 0.015 |  |
|  | VH deep: Small *H. crassispina* in crevices | 0.020 |  |
|  | VH deep: Large *H. crassispina* in crevices | 0.024 |  |
|  | VH deep: Large *H. crassispina* as free-living | 0.021 |  |
|  | VH shallow: Small *D. savignyi* in pits | 0.021 |  |
|  | VH shallow: Medium *D. savignyi* in crevices | 0.022 |  |
|  | VH shallow: Large *D. savignyi* in crevices | 0.026 |  |
|  | VH shallow: Medium *D. savignyi* as free-living | 0.019 |  |
|  | VH shallow: Large *D. savignyi* as free-living | 0.023 |  |
|  | VH shallow: Small *D.* *setosum* in pits | 0.015 |  |
|  | VH shallow: Medium *D. setosum* in pits | 0.016 |  |
|  | VH shallow: Small *D. setosum* in crevices | 0.016 |  |
|  | VH shallow: Medium *D. setosum* in crevices | 0.017 |  |
|  | VH shallow: Large *D. setosum* in crevices | 0.020 |  |
|  | VH shallow: Medium *D. setosum* as free-living | 0.014 |  |
|  | VH shallow: Large *D. setosum* as free-living | 0.017 |  |
|  | VH shallow: Small *H. crassispina* in pits | 0.022 |  |
|  | VH shallow: Medium *H. crassispina* in pits | 0.023 |  |
|  | VH shallow: Medium *H. crassispina* in crevices | 0.023 |  |
|  | VH shallow: Medium *H. crassispina* as free-living | 0.020 |  |
| D. savignyi density | IH deep | 0.161 | Hurdle negative binomial |
|  | IH shallow | 0.230 |  |
|  | VH deep | 0.270 |  |
|  | VH shallow | 0.339 |  |
| D. setosum density | IH deep | 0.263 | Hurdle negative binomial |
|  | IH shallow | 0.235 |  |
|  | VH deep | 0.265 |  |
|  | VH shallow | 0.237 |  |
| H. crassispina density | IH deep | 0.112 | Hurdle negative binomial |
|  | IH shallow | 0.293 |  |
|  | VH deep | 0.207 |  |
|  | VH shallow | 0.388 |  |
| *D. savignyi* biomass | IH deep | 0.230 | Hurdle Gamma |
|  | IH shallow | 0.231 |  |
|  | VH deep | 0.269 |  |
|  | VH shallow | 0.270 |  |
| *D. setosum* biomass | IH deep | 0.245 | Hurdle Gamma |
|  | IH shallow | 0.232 |  |
|  | VH deep | 0.268 |  |
|  | VH shallow | 0.255 |  |
| *H. crassispina* biomass | IH deep | 0.196 | Hurdle Gamma |
|  | IH shallow | 0.222 |  |
|  | VH deep | 0.278 |  |
|  | VH shallow | 0.304 |  |
| Linear displacement | IH: *D. setosum* at 1st recapture | 0.130 | Hurdle Gamma |
|  | IH: *D. setosum* at 2nd recapture | 0.203 |  |
|  | IH: *H. crassispina* at 1st recapture | 0.120 |  |
|  | IH: *H. crassispina* at 2nd recapture | 0.047 |  |
|  | VH: *D. setosum* at 1st recapture | 0.070 |  |
|  | VH: *D. setosum* at 2nd recapture | 0.143 |  |
|  | VH: *H. crassispina* at 1st recapture | 0.180 |  |
|  | VH: *H. crassispina* at 2nd recapture | 0.107 |  |
| Group composition | IH: *D. setosum* at start | 0.191 | Negative binomial |
|  | IH: *D. setosum* at 1st recapture | 0.120 |  |
|  | IH: *D. setosum* at 2nd recapture | 0.132 |  |
|  | VH: *D. setosum* at start | 0.229 |  |
|  | VH: *D. setosum* at 1st recapture | 0.158 |  |
|  | VH: *D. setosum* at 2nd recapture | 0.170 |  |
