# Supplementary material for: The behavior of sympatric sea urchin species across an ecosystem state gradient
Source: PeerJ. 2023 Jun 13;11:e15511. doi: 10.7717/peerj.15511 (PMC10274604; doi:10.7717/peerj.15511)
Supplement: Supplemental Information 8 — The mean and 95% highest density credible interval for the expectations of the model on the sea urchin density (indiv. m−2) in the deep and shallow transects of the isoyake and vegetated habitat. [file peerj-11-15511-s008.docx]

| **Month** | **Habitat** | **Transect** | **Species** | **Urchin density (indiv. m^-2^) GAM** | | |
| --- | --- | --- | --- | --- | --- | --- |
|  |  |  |  | **Mean** | **Lower** | **Upper** |
| 2020-Sep | Isoyake | Deep | *D. savignyi* | 0.09 | 0.00 | 0.24 |
| 2020-Oct | Isoyake | Deep | *D. savignyi* | 0.09 | 0.00 | 0.23 |
| 2020-Nov | Isoyake | Deep | *D. savignyi* | 0.09 | 0.00 | 0.22 |
| 2020-Dec | Isoyake | Deep | *D. savignyi* | 0.09 | 0.01 | 0.22 |
| 2021-Jan | Isoyake | Deep | *D. savignyi* | 0.10 | 0.02 | 0.21 |
| 2021-Feb | Isoyake | Deep | *D. savignyi* | 0.11 | 0.03 | 0.21 |
| 2021-Mar | Isoyake | Deep | *D. savignyi* | 0.12 | 0.05 | 0.21 |
| 2021-Apr | Isoyake | Deep | *D. savignyi* | 0.12 | 0.06 | 0.21 |
| 2021-May | Isoyake | Deep | *D. savignyi* | 0.13 | 0.06 | 0.20 |
| 2021-Jun | Isoyake | Deep | *D. savignyi* | 0.13 | 0.06 | 0.20 |
| 2021-Jul | Isoyake | Deep | *D. savignyi* | 0.12 | 0.06 | 0.19 |
| 2021-Aug | Isoyake | Deep | *D. savignyi* | 0.12 | 0.06 | 0.19 |
| 2021-Sep | Isoyake | Deep | *D. savignyi* | 0.11 | 0.05 | 0.17 |
| 2021-Oct | Isoyake | Deep | *D. savignyi* | 0.09 | 0.04 | 0.16 |
| 2021-Nov | Isoyake | Deep | *D. savignyi* | 0.08 | 0.02 | 0.14 |
| 2021-Dec | Isoyake | Deep | *D. savignyi* | 0.07 | 0.01 | 0.13 |
| 2020-Sep | Isoyake | Deep | *D. setosum* | 1.22 | 0.59 | 2.05 |
| 2020-Oct | Isoyake | Deep | *D. setosum* | 1.23 | 0.70 | 1.87 |
| 2020-Nov | Isoyake | Deep | *D. setosum* | 1.26 | 0.74 | 1.81 |
| 2020-Dec | Isoyake | Deep | *D. setosum* | 1.26 | 0.76 | 1.84 |
| 2021-Jan | Isoyake | Deep | *D. setosum* | 1.21 | 0.72 | 1.75 |
| 2021-Feb | Isoyake | Deep | *D. setosum* | 1.11 | 0.68 | 1.55 |
| 2021-Mar | Isoyake | Deep | *D. setosum* | 0.99 | 0.63 | 1.38 |
| 2021-Apr | Isoyake | Deep | *D. setosum* | 0.89 | 0.55 | 1.27 |
| 2021-May | Isoyake | Deep | *D. setosum* | 0.85 | 0.49 | 1.24 |
| 2021-Jun | Isoyake | Deep | *D. setosum* | 0.86 | 0.51 | 1.28 |
| 2021-Jul | Isoyake | Deep | *D. setosum* | 0.92 | 0.53 | 1.36 |
| 2021-Aug | Isoyake | Deep | *D. setosum* | 1.02 | 0.55 | 1.51 |
| 2021-Sep | Isoyake | Deep | *D. setosum* | 1.15 | 0.66 | 1.69 |
| 2021-Oct | Isoyake | Deep | *D. setosum* | 1.27 | 0.74 | 1.83 |
| 2021-Nov | Isoyake | Deep | *D. setosum* | 1.38 | 0.81 | 2.07 |
| 2021-Dec | Isoyake | Deep | *D. setosum* | 1.48 | 0.70 | 2.33 |
| 2020-Sep | Isoyake | Deep | *H. crassispina* | 0.17 | 0.08 | 0.30 |
| 2020-Oct | Isoyake | Deep | *H. crassispina* | 0.17 | 0.09 | 0.27 |
| 2020-Nov | Isoyake | Deep | *H. crassispina* | 0.17 | 0.10 | 0.26 |
| 2020-Dec | Isoyake | Deep | *H. crassispina* | 0.17 | 0.10 | 0.25 |
| 2021-Jan | Isoyake | Deep | *H. crassispina* | 0.16 | 0.09 | 0.24 |
| 2021-Feb | Isoyake | Deep | *H. crassispina* | 0.16 | 0.10 | 0.24 |
| 2021-Mar | Isoyake | Deep | *H. crassispina* | 0.15 | 0.09 | 0.22 |
| 2021-Apr | Isoyake | Deep | *H. crassispina* | 0.14 | 0.09 | 0.21 |
| 2021-May | Isoyake | Deep | *H. crassispina* | 0.13 | 0.07 | 0.20 |
| 2021-Jun | Isoyake | Deep | *H. crassispina* | 0.11 | 0.06 | 0.17 |
| 2021-Jul | Isoyake | Deep | *H. crassispina* | 0.09 | 0.03 | 0.15 |
| 2021-Aug | Isoyake | Deep | *H. crassispina* | 0.08 | 0.02 | 0.14 |
| 2021-Sep | Isoyake | Deep | *H. crassispina* | 0.07 | 0.01 | 0.13 |
| 2021-Oct | Isoyake | Deep | *H. crassispina* | 0.06 | 0.00 | 0.13 |
| 2021-Nov | Isoyake | Deep | *H. crassispina* | 0.07 | 0.00 | 0.14 |
| 2021-Dec | Isoyake | Deep | *H. crassispina* | 0.07 | 0.00 | 0.17 |
| 2020-Sep | Isoyake | Shallow | *D. savignyi* | 0.11 | 0.02 | 0.22 |
| 2020-Oct | Isoyake | Shallow | *D. savignyi* | 0.12 | 0.03 | 0.21 |
| 2020-Nov | Isoyake | Shallow | *D. savignyi* | 0.13 | 0.05 | 0.22 |
| 2020-Dec | Isoyake | Shallow | *D. savignyi* | 0.14 | 0.06 | 0.24 |
| 2021-Jan | Isoyake | Shallow | *D. savignyi* | 0.15 | 0.06 | 0.26 |
| 2021-Feb | Isoyake | Shallow | *D. savignyi* | 0.16 | 0.07 | 0.27 |
| 2021-Mar | Isoyake | Shallow | *D. savignyi* | 0.17 | 0.08 | 0.27 |
| 2021-Apr | Isoyake | Shallow | *D. savignyi* | 0.17 | 0.08 | 0.27 |
| 2021-May | Isoyake | Shallow | *D. savignyi* | 0.17 | 0.09 | 0.27 |
| 2021-Jun | Isoyake | Shallow | *D. savignyi* | 0.17 | 0.08 | 0.26 |
| 2021-Jul | Isoyake | Shallow | *D. savignyi* | 0.16 | 0.08 | 0.25 |
| 2021-Aug | Isoyake | Shallow | *D. savignyi* | 0.15 | 0.08 | 0.24 |
| 2021-Sep | Isoyake | Shallow | *D. savignyi* | 0.13 | 0.06 | 0.21 |
| 2021-Oct | Isoyake | Shallow | *D. savignyi* | 0.12 | 0.05 | 0.20 |
| 2021-Nov | Isoyake | Shallow | *D. savignyi* | 0.10 | 0.03 | 0.18 |
| 2021-Dec | Isoyake | Shallow | *D. savignyi* | 0.08 | 0.01 | 0.17 |
| 2020-Sep | Isoyake | Shallow | *D. setosum* | 1.11 | 0.55 | 1.78 |
| 2020-Oct | Isoyake | Shallow | *D. setosum* | 1.20 | 0.70 | 1.80 |
| 2020-Nov | Isoyake | Shallow | *D. setosum* | 1.28 | 0.77 | 1.88 |
| 2020-Dec | Isoyake | Shallow | *D. setosum* | 1.32 | 0.76 | 1.97 |
| 2021-Jan | Isoyake | Shallow | *D. setosum* | 1.31 | 0.75 | 1.97 |
| 2021-Feb | Isoyake | Shallow | *D. setosum* | 1.25 | 0.76 | 1.83 |
| 2021-Mar | Isoyake | Shallow | *D. setosum* | 1.15 | 0.73 | 1.62 |
| 2021-Apr | Isoyake | Shallow | *D. setosum* | 1.06 | 0.68 | 1.50 |
| 2021-May | Isoyake | Shallow | *D. setosum* | 1.02 | 0.61 | 1.45 |
| 2021-Jun | Isoyake | Shallow | *D. setosum* | 1.03 | 0.62 | 1.44 |
| 2021-Jul | Isoyake | Shallow | *D. setosum* | 1.09 | 0.70 | 1.50 |
| 2021-Aug | Isoyake | Shallow | *D. setosum* | 1.17 | 0.74 | 1.62 |
| 2021-Sep | Isoyake | Shallow | *D. setosum* | 1.22 | 0.75 | 1.70 |
| 2021-Oct | Isoyake | Shallow | *D. setosum* | 1.22 | 0.76 | 1.75 |
| 2021-Nov | Isoyake | Shallow | *D. setosum* | 1.19 | 0.64 | 1.81 |
| 2021-Dec | Isoyake | Shallow | *D. setosum* | 1.16 | 0.47 | 1.95 |
| 2020-Sep | Isoyake | Shallow | *H. crassispina* | 0.71 | 0.31 | 1.16 |
| 2020-Oct | Isoyake | Shallow | *H. crassispina* | 0.72 | 0.39 | 1.07 |
| 2020-Nov | Isoyake | Shallow | *H. crassispina* | 0.74 | 0.43 | 1.05 |
| 2020-Dec | Isoyake | Shallow | *H. crassispina* | 0.78 | 0.47 | 1.10 |
| 2021-Jan | Isoyake | Shallow | *H. crassispina* | 0.81 | 0.49 | 1.16 |
| 2021-Feb | Isoyake | Shallow | *H. crassispina* | 0.83 | 0.51 | 1.18 |
| 2021-Mar | Isoyake | Shallow | *H. crassispina* | 0.82 | 0.53 | 1.16 |
| 2021-Apr | Isoyake | Shallow | *H. crassispina* | 0.78 | 0.50 | 1.09 |
| 2021-May | Isoyake | Shallow | *H. crassispina* | 0.72 | 0.45 | 0.99 |
| 2021-Jun | Isoyake | Shallow | *H. crassispina* | 0.62 | 0.40 | 0.86 |
| 2021-Jul | Isoyake | Shallow | *H. crassispina* | 0.52 | 0.33 | 0.74 |
| 2021-Aug | Isoyake | Shallow | *H. crassispina* | 0.45 | 0.24 | 0.65 |
| 2021-Sep | Isoyake | Shallow | *H. crassispina* | 0.41 | 0.21 | 0.63 |
| 2021-Oct | Isoyake | Shallow | *H. crassispina* | 0.42 | 0.22 | 0.63 |
| 2021-Nov | Isoyake | Shallow | *H. crassispina* | 0.45 | 0.24 | 0.68 |
| 2021-Dec | Isoyake | Shallow | *H. crassispina* | 0.51 | 0.23 | 0.85 |
| 2020-Sep | Vegetated | Deep | *D. savignyi* | 0.12 | 0.02 | 0.23 |
| 2020-Oct | Vegetated | Deep | *D. savignyi* | 0.13 | 0.04 | 0.23 |
| 2020-Nov | Vegetated | Deep | *D. savignyi* | 0.14 | 0.05 | 0.24 |
| 2020-Dec | Vegetated | Deep | *D. savignyi* | 0.16 | 0.06 | 0.26 |
| 2021-Jan | Vegetated | Deep | *D. savignyi* | 0.17 | 0.07 | 0.29 |
| 2021-Feb | Vegetated | Deep | *D. savignyi* | 0.19 | 0.09 | 0.30 |
| 2021-Mar | Vegetated | Deep | *D. savignyi* | 0.21 | 0.11 | 0.33 |
| 2021-Apr | Vegetated | Deep | *D. savignyi* | 0.24 | 0.13 | 0.36 |
| 2021-May | Vegetated | Deep | *D. savignyi* | 0.26 | 0.14 | 0.38 |
| 2021-Jun | Vegetated | Deep | *D. savignyi* | 0.27 | 0.16 | 0.40 |
| 2021-Jul | Vegetated | Deep | *D. savignyi* | 0.28 | 0.15 | 0.40 |
| 2021-Aug | Vegetated | Deep | *D. savignyi* | 0.27 | 0.15 | 0.40 |
| 2021-Sep | Vegetated | Deep | *D. savignyi* | 0.24 | 0.13 | 0.37 |
| 2021-Oct | Vegetated | Deep | *D. savignyi* | 0.21 | 0.11 | 0.33 |
| 2021-Nov | Vegetated | Deep | *D. savignyi* | 0.18 | 0.08 | 0.31 |
| 2021-Dec | Vegetated | Deep | *D. savignyi* | 0.15 | 0.04 | 0.29 |
| 2020-Sep | Vegetated | Deep | *D. setosum* | 2.76 | 1.35 | 4.48 |
| 2020-Oct | Vegetated | Deep | *D. setosum* | 2.77 | 1.57 | 4.03 |
| 2020-Nov | Vegetated | Deep | *D. setosum* | 2.77 | 1.72 | 3.95 |
| 2020-Dec | Vegetated | Deep | *D. setosum* | 2.71 | 1.68 | 3.91 |
| 2021-Jan | Vegetated | Deep | *D. setosum* | 2.55 | 1.52 | 3.62 |
| 2021-Feb | Vegetated | Deep | *D. setosum* | 2.29 | 1.48 | 3.26 |
| 2021-Mar | Vegetated | Deep | *D. setosum* | 2.03 | 1.29 | 2.78 |
| 2021-Apr | Vegetated | Deep | *D. setosum* | 1.83 | 1.13 | 2.55 |
| 2021-May | Vegetated | Deep | *D. setosum* | 1.74 | 1.09 | 2.50 |
| 2021-Jun | Vegetated | Deep | *D. setosum* | 1.77 | 1.10 | 2.51 |
| 2021-Jul | Vegetated | Deep | *D. setosum* | 1.90 | 1.21 | 2.68 |
| 2021-Aug | Vegetated | Deep | *D. setosum* | 2.11 | 1.31 | 2.97 |
| 2021-Sep | Vegetated | Deep | *D. setosum* | 2.35 | 1.42 | 3.25 |
| 2021-Oct | Vegetated | Deep | *D. setosum* | 2.57 | 1.67 | 3.63 |
| 2021-Nov | Vegetated | Deep | *D. setosum* | 2.76 | 1.65 | 3.97 |
| 2021-Dec | Vegetated | Deep | *D. setosum* | 2.95 | 1.53 | 4.67 |
| 2020-Sep | Vegetated | Deep | *H. crassispina* | 0.16 | 0.04 | 0.30 |
| 2020-Oct | Vegetated | Deep | *H. crassispina* | 0.17 | 0.07 | 0.29 |
| 2020-Nov | Vegetated | Deep | *H. crassispina* | 0.17 | 0.08 | 0.28 |
| 2020-Dec | Vegetated | Deep | *H. crassispina* | 0.18 | 0.09 | 0.28 |
| 2021-Jan | Vegetated | Deep | *H. crassispina* | 0.19 | 0.10 | 0.28 |
| 2021-Feb | Vegetated | Deep | *H. crassispina* | 0.19 | 0.11 | 0.28 |
| 2021-Mar | Vegetated | Deep | *H. crassispina* | 0.19 | 0.12 | 0.28 |
| 2021-Apr | Vegetated | Deep | *H. crassispina* | 0.19 | 0.12 | 0.28 |
| 2021-May | Vegetated | Deep | *H. crassispina* | 0.19 | 0.11 | 0.27 |
| 2021-Jun | Vegetated | Deep | *H. crassispina* | 0.17 | 0.11 | 0.25 |
| 2021-Jul | Vegetated | Deep | *H. crassispina* | 0.16 | 0.10 | 0.23 |
| 2021-Aug | Vegetated | Deep | *H. crassispina* | 0.15 | 0.09 | 0.23 |
| 2021-Sep | Vegetated | Deep | *H. crassispina* | 0.15 | 0.09 | 0.22 |
| 2021-Oct | Vegetated | Deep | *H. crassispina* | 0.16 | 0.09 | 0.24 |
| 2021-Nov | Vegetated | Deep | *H. crassispina* | 0.17 | 0.09 | 0.26 |
| 2021-Dec | Vegetated | Deep | *H. crassispina* | 0.19 | 0.08 | 0.33 |
| 2020-Sep | Vegetated | Shallow | *D. savignyi* | 0.15 | 0.02 | 0.30 |
| 2020-Oct | Vegetated | Shallow | *D. savignyi* | 0.15 | 0.04 | 0.29 |
| 2020-Nov | Vegetated | Shallow | *D. savignyi* | 0.17 | 0.06 | 0.29 |
| 2020-Dec | Vegetated | Shallow | *D. savignyi* | 0.19 | 0.08 | 0.32 |
| 2021-Jan | Vegetated | Shallow | *D. savignyi* | 0.21 | 0.09 | 0.35 |
| 2021-Feb | Vegetated | Shallow | *D. savignyi* | 0.24 | 0.11 | 0.39 |
| 2021-Mar | Vegetated | Shallow | *D. savignyi* | 0.28 | 0.15 | 0.44 |
| 2021-Apr | Vegetated | Shallow | *D. savignyi* | 0.33 | 0.18 | 0.50 |
| 2021-May | Vegetated | Shallow | *D. savignyi* | 0.39 | 0.21 | 0.58 |
| 2021-Jun | Vegetated | Shallow | *D. savignyi* | 0.44 | 0.25 | 0.66 |
| 2021-Jul | Vegetated | Shallow | *D. savignyi* | 0.49 | 0.28 | 0.72 |
| 2021-Aug | Vegetated | Shallow | *D. savignyi* | 0.50 | 0.29 | 0.76 |
| 2021-Sep | Vegetated | Shallow | *D. savignyi* | 0.48 | 0.26 | 0.71 |
| 2021-Oct | Vegetated | Shallow | *D. savignyi* | 0.43 | 0.23 | 0.66 |
| 2021-Nov | Vegetated | Shallow | *D. savignyi* | 0.37 | 0.17 | 0.60 |
| 2021-Dec | Vegetated | Shallow | *D. savignyi* | 0.32 | 0.10 | 0.59 |
| 2020-Sep | Vegetated | Shallow | *D. setosum* | 0.37 | 0.15 | 0.63 |
| 2020-Oct | Vegetated | Shallow | *D. setosum* | 0.40 | 0.20 | 0.63 |
| 2020-Nov | Vegetated | Shallow | *D. setosum* | 0.42 | 0.23 | 0.65 |
| 2020-Dec | Vegetated | Shallow | *D. setosum* | 0.43 | 0.22 | 0.67 |
| 2021-Jan | Vegetated | Shallow | *D. setosum* | 0.42 | 0.20 | 0.66 |
| 2021-Feb | Vegetated | Shallow | *D. setosum* | 0.39 | 0.20 | 0.61 |
| 2021-Mar | Vegetated | Shallow | *D. setosum* | 0.37 | 0.19 | 0.56 |
| 2021-Apr | Vegetated | Shallow | *D. setosum* | 0.35 | 0.18 | 0.52 |
| 2021-May | Vegetated | Shallow | *D. setosum* | 0.35 | 0.20 | 0.53 |
| 2021-Jun | Vegetated | Shallow | *D. setosum* | 0.38 | 0.23 | 0.56 |
| 2021-Jul | Vegetated | Shallow | *D. setosum* | 0.42 | 0.25 | 0.61 |
| 2021-Aug | Vegetated | Shallow | *D. setosum* | 0.45 | 0.27 | 0.67 |
| 2021-Sep | Vegetated | Shallow | *D. setosum* | 0.47 | 0.28 | 0.69 |
| 2021-Oct | Vegetated | Shallow | *D. setosum* | 0.47 | 0.27 | 0.71 |
| 2021-Nov | Vegetated | Shallow | *D. setosum* | 0.47 | 0.22 | 0.73 |
| 2021-Dec | Vegetated | Shallow | *D. setosum* | 0.47 | 0.18 | 0.80 |
| 2020-Sep | Vegetated | Shallow | *H. crassispina* | 1.99 | 0.92 | 3.13 |
| 2020-Oct | Vegetated | Shallow | *H. crassispina* | 2.13 | 1.26 | 3.04 |
| 2020-Nov | Vegetated | Shallow | *H. crassispina* | 2.26 | 1.48 | 3.10 |
| 2020-Dec | Vegetated | Shallow | *H. crassispina* | 2.39 | 1.59 | 3.25 |
| 2021-Jan | Vegetated | Shallow | *H. crassispina* | 2.51 | 1.65 | 3.41 |
| 2021-Feb | Vegetated | Shallow | *H. crassispina* | 2.63 | 1.83 | 3.61 |
| 2021-Mar | Vegetated | Shallow | *H. crassispina* | 2.72 | 1.88 | 3.66 |
| 2021-Apr | Vegetated | Shallow | *H. crassispina* | 2.76 | 1.90 | 3.77 |
| 2021-May | Vegetated | Shallow | *H. crassispina* | 2.72 | 1.85 | 3.72 |
| 2021-Jun | Vegetated | Shallow | *H. crassispina* | 2.61 | 1.79 | 3.48 |
| 2021-Jul | Vegetated | Shallow | *H. crassispina* | 2.50 | 1.72 | 3.29 |
| 2021-Aug | Vegetated | Shallow | *H. crassispina* | 2.44 | 1.63 | 3.30 |
| 2021-Sep | Vegetated | Shallow | *H. crassispina* | 2.47 | 1.62 | 3.35 |
| 2021-Oct | Vegetated | Shallow | *H. crassispina* | 2.59 | 1.70 | 3.48 |
| 2021-Nov | Vegetated | Shallow | *H. crassispina* | 2.78 | 1.76 | 3.99 |
| 2021-Dec | Vegetated | Shallow | *H. crassispina* | 3.02 | 1.49 | 5.00 |
