# Supplementary material for: The behavior of sympatric sea urchin species across an ecosystem state gradient
Source: PeerJ. 2023 Jun 13;11:e15511. doi: 10.7717/peerj.15511 (PMC10274604; doi:10.7717/peerj.15511)
Supplement: Supplemental Information 9 — The mean and 95% highest density credible interval for the expectations of the model on the sea urchin biomass (grams m−2) in the deep and shallow transects of the isoyake and vegetated habitat. [file peerj-11-15511-s009.docx]

| **Month** | **Habitat** | **Transect** | **Species** | **Urchin biomass (g m^-2^) GAM** | | |
| --- | --- | --- | --- | --- | --- | --- |
|  |  |  |  | **Mean** | **Lower** | **Upper** |
| 2020-Sep | Isoyake | Deep | *D. savignyi* | 2.68 | 0.43 | 5.41 |
| 2020-Oct | Isoyake | Deep | *D. savignyi* | 2.70 | 0.77 | 4.89 |
| 2020-Nov | Isoyake | Deep | *D. savignyi* | 2.76 | 0.93 | 4.75 |
| 2020-Dec | Isoyake | Deep | *D. savignyi* | 2.84 | 1.04 | 4.91 |
| 2021-Jan | Isoyake | Deep | *D. savignyi* | 2.95 | 1.13 | 4.99 |
| 2021-Feb | Isoyake | Deep | *D. savignyi* | 3.09 | 1.40 | 5.04 |
| 2021-Mar | Isoyake | Deep | *D. savignyi* | 3.23 | 1.64 | 5.04 |
| 2021-Apr | Isoyake | Deep | *D. savignyi* | 3.31 | 1.68 | 5.05 |
| 2021-May | Isoyake | Deep | *D. savignyi* | 3.31 | 1.71 | 5.03 |
| 2021-Jun | Isoyake | Deep | *D. savignyi* | 3.21 | 1.65 | 4.86 |
| 2021-Jul | Isoyake | Deep | *D. savignyi* | 3.10 | 1.56 | 4.78 |
| 2021-Aug | Isoyake | Deep | *D. savignyi* | 3.06 | 1.50 | 4.80 |
| 2021-Sep | Isoyake | Deep | *D. savignyi* | 3.13 | 1.48 | 4.88 |
| 2021-Oct | Isoyake | Deep | *D. savignyi* | 3.32 | 1.63 | 5.09 |
| 2021-Nov | Isoyake | Deep | *D. savignyi* | 3.58 | 1.64 | 5.75 |
| 2021-Dec | Isoyake | Deep | *D. savignyi* | 3.85 | 0.89 | 7.05 |
| 2020-Sep | Isoyake | Deep | *D. setosum* | 4.52 | 3.37 | 5.79 |
| 2020-Oct | Isoyake | Deep | *D. setosum* | 4.38 | 3.39 | 5.31 |
| 2020-Nov | Isoyake | Deep | *D. setosum* | 4.22 | 3.33 | 5.07 |
| 2020-Dec | Isoyake | Deep | *D. setosum* | 4.04 | 3.20 | 4.95 |
| 2021-Jan | Isoyake | Deep | *D. setosum* | 3.86 | 2.98 | 4.77 |
| 2021-Feb | Isoyake | Deep | *D. setosum* | 3.70 | 2.84 | 4.50 |
| 2021-Mar | Isoyake | Deep | *D. setosum* | 3.56 | 2.78 | 4.32 |
| 2021-Apr | Isoyake | Deep | *D. setosum* | 3.47 | 2.70 | 4.23 |
| 2021-May | Isoyake | Deep | *D. setosum* | 3.42 | 2.70 | 4.24 |
| 2021-Jun | Isoyake | Deep | *D. setosum* | 3.45 | 2.72 | 4.21 |
| 2021-Jul | Isoyake | Deep | *D. setosum* | 3.52 | 2.78 | 4.27 |
| 2021-Aug | Isoyake | Deep | *D. setosum* | 3.62 | 2.85 | 4.42 |
| 2021-Sep | Isoyake | Deep | *D. setosum* | 3.73 | 2.97 | 4.58 |
| 2021-Oct | Isoyake | Deep | *D. setosum* | 3.82 | 3.05 | 4.64 |
| 2021-Nov | Isoyake | Deep | *D. setosum* | 3.89 | 3.01 | 4.72 |
| 2021-Dec | Isoyake | Deep | *D. setosum* | 3.94 | 2.92 | 5.02 |
| 2020-Sep | Isoyake | Deep | *H. crassispina* | 1.56 | 0.87 | 2.17 |
| 2020-Oct | Isoyake | Deep | *H. crassispina* | 1.61 | 1.12 | 2.11 |
| 2020-Nov | Isoyake | Deep | *H. crassispina* | 1.62 | 1.19 | 2.07 |
| 2020-Dec | Isoyake | Deep | *H. crassispina* | 1.63 | 1.21 | 2.08 |
| 2021-Jan | Isoyake | Deep | *H. crassispina* | 1.63 | 1.20 | 2.06 |
| 2021-Feb | Isoyake | Deep | *H. crassispina* | 1.62 | 1.22 | 2.05 |
| 2021-Mar | Isoyake | Deep | *H. crassispina* | 1.61 | 1.19 | 2.01 |
| 2021-Apr | Isoyake | Deep | *H. crassispina* | 1.59 | 1.15 | 2.00 |
| 2021-May | Isoyake | Deep | *H. crassispina* | 1.56 | 1.15 | 2.02 |
| 2021-Jun | Isoyake | Deep | *H. crassispina* | 1.53 | 1.13 | 1.99 |
| 2021-Jul | Isoyake | Deep | *H. crassispina* | 1.49 | 1.07 | 1.95 |
| 2021-Aug | Isoyake | Deep | *H. crassispina* | 1.44 | 0.97 | 1.89 |
| 2021-Sep | Isoyake | Deep | *H. crassispina* | 1.40 | 0.95 | 1.89 |
| 2021-Oct | Isoyake | Deep | *H. crassispina* | 1.36 | 0.88 | 1.82 |
| 2021-Nov | Isoyake | Deep | *H. crassispina* | 1.32 | 0.80 | 1.81 |
| 2021-Dec | Isoyake | Deep | *H. crassispina* | 1.27 | 0.68 | 1.90 |
| 2020-Sep | Isoyake | Shallow | *D. savignyi* | 2.85 | 0.51 | 5.16 |
| 2020-Oct | Isoyake | Shallow | *D. savignyi* | 2.84 | 1.04 | 4.71 |
| 2020-Nov | Isoyake | Shallow | *D. savignyi* | 2.86 | 1.28 | 4.58 |
| 2020-Dec | Isoyake | Shallow | *D. savignyi* | 2.90 | 1.34 | 4.65 |
| 2021-Jan | Isoyake | Shallow | *D. savignyi* | 2.97 | 1.36 | 4.61 |
| 2021-Feb | Isoyake | Shallow | *D. savignyi* | 3.06 | 1.53 | 4.58 |
| 2021-Mar | Isoyake | Shallow | *D. savignyi* | 3.15 | 1.77 | 4.63 |
| 2021-Apr | Isoyake | Shallow | *D. savignyi* | 3.20 | 1.86 | 4.73 |
| 2021-May | Isoyake | Shallow | *D. savignyi* | 3.16 | 1.82 | 4.64 |
| 2021-Jun | Isoyake | Shallow | *D. savignyi* | 3.05 | 1.85 | 4.42 |
| 2021-Jul | Isoyake | Shallow | *D. savignyi* | 2.93 | 1.74 | 4.20 |
| 2021-Aug | Isoyake | Shallow | *D. savignyi* | 2.88 | 1.62 | 4.20 |
| 2021-Sep | Isoyake | Shallow | *D. savignyi* | 2.97 | 1.74 | 4.41 |
| 2021-Oct | Isoyake | Shallow | *D. savignyi* | 3.21 | 1.84 | 4.69 |
| 2021-Nov | Isoyake | Shallow | *D. savignyi* | 3.54 | 1.61 | 5.45 |
| 2021-Dec | Isoyake | Shallow | *D. savignyi* | 3.92 | 1.01 | 7.13 |
| 2020-Sep | Isoyake | Shallow | *D. setosum* | 4.17 | 3.17 | 5.33 |
| 2020-Oct | Isoyake | Shallow | *D. setosum* | 4.03 | 3.21 | 4.94 |
| 2020-Nov | Isoyake | Shallow | *D. setosum* | 3.89 | 3.14 | 4.72 |
| 2020-Dec | Isoyake | Shallow | *D. setosum* | 3.73 | 2.96 | 4.57 |
| 2021-Jan | Isoyake | Shallow | *D. setosum* | 3.57 | 2.77 | 4.42 |
| 2021-Feb | Isoyake | Shallow | *D. setosum* | 3.41 | 2.68 | 4.22 |
| 2021-Mar | Isoyake | Shallow | *D. setosum* | 3.29 | 2.59 | 4.00 |
| 2021-Apr | Isoyake | Shallow | *D. setosum* | 3.20 | 2.49 | 3.88 |
| 2021-May | Isoyake | Shallow | *D. setosum* | 3.16 | 2.45 | 3.86 |
| 2021-Jun | Isoyake | Shallow | *D. setosum* | 3.18 | 2.48 | 3.85 |
| 2021-Jul | Isoyake | Shallow | *D. setosum* | 3.25 | 2.59 | 3.94 |
| 2021-Aug | Isoyake | Shallow | *D. setosum* | 3.34 | 2.65 | 4.07 |
| 2021-Sep | Isoyake | Shallow | *D. setosum* | 3.44 | 2.77 | 4.20 |
| 2021-Oct | Isoyake | Shallow | *D. setosum* | 3.52 | 2.83 | 4.24 |
| 2021-Nov | Isoyake | Shallow | *D. setosum* | 3.58 | 2.88 | 4.42 |
| 2021-Dec | Isoyake | Shallow | *D. setosum* | 3.63 | 2.72 | 4.69 |
| 2020-Sep | Isoyake | Shallow | *H. crassispina* | 2.58 | 1.75 | 3.51 |
| 2020-Oct | Isoyake | Shallow | *H. crassispina* | 2.61 | 1.93 | 3.29 |
| 2020-Nov | Isoyake | Shallow | *H. crassispina* | 2.62 | 2.02 | 3.24 |
| 2020-Dec | Isoyake | Shallow | *H. crassispina* | 2.62 | 2.05 | 3.22 |
| 2021-Jan | Isoyake | Shallow | *H. crassispina* | 2.61 | 2.05 | 3.21 |
| 2021-Feb | Isoyake | Shallow | *H. crassispina* | 2.60 | 2.07 | 3.18 |
| 2021-Mar | Isoyake | Shallow | *H. crassispina* | 2.58 | 2.06 | 3.14 |
| 2021-Apr | Isoyake | Shallow | *H. crassispina* | 2.56 | 2.04 | 3.13 |
| 2021-May | Isoyake | Shallow | *H. crassispina* | 2.53 | 1.96 | 3.06 |
| 2021-Jun | Isoyake | Shallow | *H. crassispina* | 2.49 | 1.97 | 3.09 |
| 2021-Jul | Isoyake | Shallow | *H. crassispina* | 2.45 | 1.87 | 3.00 |
| 2021-Aug | Isoyake | Shallow | *H. crassispina* | 2.40 | 1.84 | 3.03 |
| 2021-Sep | Isoyake | Shallow | *H. crassispina* | 2.34 | 1.77 | 3.01 |
| 2021-Oct | Isoyake | Shallow | *H. crassispina* | 2.29 | 1.71 | 2.93 |
| 2021-Nov | Isoyake | Shallow | *H. crassispina* | 2.24 | 1.56 | 2.88 |
| 2021-Dec | Isoyake | Shallow | *H. crassispina* | 2.17 | 1.37 | 2.97 |
| 2020-Sep | Vegetated | Deep | *D. savignyi* | 5.61 | 1.80 | 10.15 |
| 2020-Oct | Vegetated | Deep | *D. savignyi* | 5.52 | 2.31 | 8.76 |
| 2020-Nov | Vegetated | Deep | *D. savignyi* | 5.46 | 2.67 | 8.31 |
| 2020-Dec | Vegetated | Deep | *D. savignyi* | 5.42 | 2.67 | 8.20 |
| 2021-Jan | Vegetated | Deep | *D. savignyi* | 5.44 | 2.96 | 8.35 |
| 2021-Feb | Vegetated | Deep | *D. savignyi* | 5.51 | 3.16 | 7.95 |
| 2021-Mar | Vegetated | Deep | *D. savignyi* | 5.58 | 3.47 | 7.80 |
| 2021-Apr | Vegetated | Deep | *D. savignyi* | 5.59 | 3.57 | 7.86 |
| 2021-May | Vegetated | Deep | *D. savignyi* | 5.48 | 3.48 | 7.69 |
| 2021-Jun | Vegetated | Deep | *D. savignyi* | 5.26 | 3.44 | 7.29 |
| 2021-Jul | Vegetated | Deep | *D. savignyi* | 5.05 | 3.16 | 6.94 |
| 2021-Aug | Vegetated | Deep | *D. savignyi* | 4.99 | 3.05 | 7.10 |
| 2021-Sep | Vegetated | Deep | *D. savignyi* | 5.19 | 3.15 | 7.43 |
| 2021-Oct | Vegetated | Deep | *D. savignyi* | 5.67 | 3.57 | 8.14 |
| 2021-Nov | Vegetated | Deep | *D. savignyi* | 6.38 | 3.31 | 9.42 |
| 2021-Dec | Vegetated | Deep | *D. savignyi* | 7.23 | 2.35 | 12.55 |
| 2020-Sep | Vegetated | Deep | *D. setosum* | 6.33 | 4.76 | 8.10 |
| 2020-Oct | Vegetated | Deep | *D. setosum* | 6.12 | 4.82 | 7.53 |
| 2020-Nov | Vegetated | Deep | *D. setosum* | 5.90 | 4.74 | 7.22 |
| 2020-Dec | Vegetated | Deep | *D. setosum* | 5.66 | 4.41 | 6.92 |
| 2021-Jan | Vegetated | Deep | *D. setosum* | 5.41 | 4.13 | 6.65 |
| 2021-Feb | Vegetated | Deep | *D. setosum* | 5.18 | 4.08 | 6.37 |
| 2021-Mar | Vegetated | Deep | *D. setosum* | 4.99 | 3.95 | 6.05 |
| 2021-Apr | Vegetated | Deep | *D. setosum* | 4.86 | 3.84 | 5.91 |
| 2021-May | Vegetated | Deep | *D. setosum* | 4.80 | 3.85 | 5.90 |
| 2021-Jun | Vegetated | Deep | *D. setosum* | 4.83 | 3.88 | 5.86 |
| 2021-Jul | Vegetated | Deep | *D. setosum* | 4.93 | 3.92 | 5.87 |
| 2021-Aug | Vegetated | Deep | *D. setosum* | 5.07 | 4.04 | 6.11 |
| 2021-Sep | Vegetated | Deep | *D. setosum* | 5.21 | 4.14 | 6.28 |
| 2021-Oct | Vegetated | Deep | *D. setosum* | 5.34 | 4.25 | 6.39 |
| 2021-Nov | Vegetated | Deep | *D. setosum* | 5.44 | 4.31 | 6.67 |
| 2021-Dec | Vegetated | Deep | *D. setosum* | 5.51 | 4.08 | 7.07 |
| 2020-Sep | Vegetated | Deep | *H. crassispina* | 3.36 | 2.06 | 4.63 |
| 2020-Oct | Vegetated | Deep | *H. crassispina* | 3.43 | 2.51 | 4.49 |
| 2020-Nov | Vegetated | Deep | *H. crassispina* | 3.46 | 2.60 | 4.34 |
| 2020-Dec | Vegetated | Deep | *H. crassispina* | 3.46 | 2.66 | 4.34 |
| 2021-Jan | Vegetated | Deep | *H. crassispina* | 3.45 | 2.66 | 4.31 |
| 2021-Feb | Vegetated | Deep | *H. crassispina* | 3.43 | 2.66 | 4.26 |
| 2021-Mar | Vegetated | Deep | *H. crassispina* | 3.40 | 2.63 | 4.17 |
| 2021-Apr | Vegetated | Deep | *H. crassispina* | 3.37 | 2.57 | 4.11 |
| 2021-May | Vegetated | Deep | *H. crassispina* | 3.33 | 2.55 | 4.10 |
| 2021-Jun | Vegetated | Deep | *H. crassispina* | 3.27 | 2.51 | 4.05 |
| 2021-Jul | Vegetated | Deep | *H. crassispina* | 3.20 | 2.40 | 3.98 |
| 2021-Aug | Vegetated | Deep | *H. crassispina* | 3.12 | 2.29 | 3.95 |
| 2021-Sep | Vegetated | Deep | *H. crassispina* | 3.04 | 2.16 | 3.88 |
| 2021-Oct | Vegetated | Deep | *H. crassispina* | 2.96 | 2.08 | 3.81 |
| 2021-Nov | Vegetated | Deep | *H. crassispina* | 2.88 | 1.95 | 3.82 |
| 2021-Dec | Vegetated | Deep | *H. crassispina* | 2.79 | 1.59 | 3.87 |
| 2020-Sep | Vegetated | Shallow | *D. savignyi* | 5.74 | 1.73 | 10.05 |
| 2020-Oct | Vegetated | Shallow | *D. savignyi* | 5.61 | 2.39 | 8.80 |
| 2020-Nov | Vegetated | Shallow | *D. savignyi* | 5.49 | 2.81 | 8.32 |
| 2020-Dec | Vegetated | Shallow | *D. savignyi* | 5.41 | 2.89 | 8.15 |
| 2021-Jan | Vegetated | Shallow | *D. savignyi* | 5.39 | 2.95 | 8.01 |
| 2021-Feb | Vegetated | Shallow | *D. savignyi* | 5.42 | 3.20 | 7.73 |
| 2021-Mar | Vegetated | Shallow | *D. savignyi* | 5.46 | 3.42 | 7.66 |
| 2021-Apr | Vegetated | Shallow | *D. savignyi* | 5.45 | 3.43 | 7.71 |
| 2021-May | Vegetated | Shallow | *D. savignyi* | 5.34 | 3.25 | 7.40 |
| 2021-Jun | Vegetated | Shallow | *D. savignyi* | 5.11 | 3.24 | 6.96 |
| 2021-Jul | Vegetated | Shallow | *D. savignyi* | 4.89 | 3.13 | 6.66 |
| 2021-Aug | Vegetated | Shallow | *D. savignyi* | 4.84 | 3.12 | 6.86 |
| 2021-Sep | Vegetated | Shallow | *D. savignyi* | 5.03 | 3.20 | 7.09 |
| 2021-Oct | Vegetated | Shallow | *D. savignyi* | 5.52 | 3.62 | 7.78 |
| 2021-Nov | Vegetated | Shallow | *D. savignyi* | 6.26 | 3.49 | 9.22 |
| 2021-Dec | Vegetated | Shallow | *D. savignyi* | 7.17 | 2.76 | 12.64 |
| 2020-Sep | Vegetated | Shallow | *D. setosum* | 4.50 | 3.31 | 5.90 |
| 2020-Oct | Vegetated | Shallow | *D. setosum* | 4.36 | 3.32 | 5.42 |
| 2020-Nov | Vegetated | Shallow | *D. setosum* | 4.20 | 3.29 | 5.24 |
| 2020-Dec | Vegetated | Shallow | *D. setosum* | 4.02 | 3.10 | 5.08 |
| 2021-Jan | Vegetated | Shallow | *D. setosum* | 3.84 | 2.85 | 4.86 |
| 2021-Feb | Vegetated | Shallow | *D. setosum* | 3.67 | 2.79 | 4.62 |
| 2021-Mar | Vegetated | Shallow | *D. setosum* | 3.53 | 2.73 | 4.41 |
| 2021-Apr | Vegetated | Shallow | *D. setosum* | 3.44 | 2.63 | 4.25 |
| 2021-May | Vegetated | Shallow | *D. setosum* | 3.40 | 2.63 | 4.22 |
| 2021-Jun | Vegetated | Shallow | *D. setosum* | 3.42 | 2.66 | 4.15 |
| 2021-Jul | Vegetated | Shallow | *D. setosum* | 3.49 | 2.74 | 4.21 |
| 2021-Aug | Vegetated | Shallow | *D. setosum* | 3.59 | 2.84 | 4.41 |
| 2021-Sep | Vegetated | Shallow | *D. setosum* | 3.70 | 2.95 | 4.54 |
| 2021-Oct | Vegetated | Shallow | *D. setosum* | 3.79 | 3.05 | 4.62 |
| 2021-Nov | Vegetated | Shallow | *D. setosum* | 3.86 | 3.03 | 4.77 |
| 2021-Dec | Vegetated | Shallow | *D. setosum* | 3.92 | 2.89 | 5.08 |
| 2020-Sep | Vegetated | Shallow | *H. crassispina* | 1.76 | 1.20 | 2.36 |
| 2020-Oct | Vegetated | Shallow | *H. crassispina* | 1.78 | 1.37 | 2.28 |
| 2020-Nov | Vegetated | Shallow | *H. crassispina* | 1.79 | 1.39 | 2.20 |
| 2020-Dec | Vegetated | Shallow | *H. crassispina* | 1.78 | 1.41 | 2.20 |
| 2021-Jan | Vegetated | Shallow | *H. crassispina* | 1.78 | 1.40 | 2.19 |
| 2021-Feb | Vegetated | Shallow | *H. crassispina* | 1.77 | 1.39 | 2.14 |
| 2021-Mar | Vegetated | Shallow | *H. crassispina* | 1.75 | 1.38 | 2.12 |
| 2021-Apr | Vegetated | Shallow | *H. crassispina* | 1.74 | 1.38 | 2.12 |
| 2021-May | Vegetated | Shallow | *H. crassispina* | 1.72 | 1.33 | 2.08 |
| 2021-Jun | Vegetated | Shallow | *H. crassispina* | 1.70 | 1.34 | 2.07 |
| 2021-Jul | Vegetated | Shallow | *H. crassispina* | 1.68 | 1.31 | 2.07 |
| 2021-Aug | Vegetated | Shallow | *H. crassispina* | 1.65 | 1.27 | 2.06 |
| 2021-Sep | Vegetated | Shallow | *H. crassispina* | 1.61 | 1.22 | 2.03 |
| 2021-Oct | Vegetated | Shallow | *H. crassispina* | 1.58 | 1.19 | 2.01 |
| 2021-Nov | Vegetated | Shallow | *H. crassispina* | 1.54 | 1.11 | 2.01 |
| 2021-Dec | Vegetated | Shallow | *H. crassispina* | 1.50 | 0.96 | 2.04 |
