# Supplementary material for: The behavior of sympatric sea urchin species across an ecosystem state gradient
Source: PeerJ. 2023 Jun 13;11:e15511. doi: 10.7717/peerj.15511 (PMC10274604; doi:10.7717/peerj.15511)
Supplement: Supplemental Information 10 — The mean expected occurrence rate and 95% highest density credible interval for the expectations of the model on the sea urchin size-class and microhabitat preference in the deep and shallow transects of the isoyake and vegetated habitat. [file peerj-11-15511-s010.docx]

| **Month** | **Habitat** | **Transect** | **Species** | **Size class** | **Microhabitat** | **Urchin microhabitat (Preference rate) GLM** | | |
| --- | --- | --- | --- | --- | --- | --- | --- | --- |
|  |  |  |  |  |  | **Mean** | **Lower** | **Upper** |
| 2020-Sep | Isoyake | Deep | *D. setosum* | Medium (4-5.9 cm) | Free-living | 80.78 | 69.41 | 92.07 |
| 2020-Sep | Isoyake | Deep | *H. crassispina* | Medium (4-5.9 cm) | Crevice | 9.23 | 2.11 | 16.83 |
| 2020-Sep | Isoyake | Deep | *H. crassispina* | Medium (4-5.9 cm) | Free-living | 13.94 | 2.74 | 28.01 |
| 2020-Sep | Isoyake | Shallow | *D. savignyi* | Large (> 6 cm) | Free-living | 6.25 | 0.77 | 13.80 |
| 2020-Sep | Isoyake | Shallow | *D. setosum* | Medium (4-5.9 cm) | Crevice | 12.83 | 3.37 | 23.71 |
| 2020-Sep | Isoyake | Shallow | *D. setosum* | Medium (4-5.9 cm) | Free-living | 51.47 | 34.90 | 68.67 |
| 2020-Sep | Isoyake | Shallow | *H. crassispina* | Medium (4-5.9 cm) | Pit | 11.71 | 2.44 | 22.90 |
| 2020-Sep | Isoyake | Shallow | *H. crassispina* | Medium (4-5.9 cm) | Crevice | 24.22 | 12.71 | 36.40 |
| 2020-Sep | Vegetated | Deep | *D. savignyi* | Large (> 6 cm) | Crevice | 7.12 | 1.09 | 14.44 |
| 2020-Sep | Vegetated | Deep | *D. setosum* | Medium (4-5.9 cm) | Crevice | 59.60 | 41.41 | 78.54 |
| 2020-Sep | Vegetated | Deep | *D. setosum* | Large (> 6 cm) | Free-living | 48.47 | 28.02 | 66.51 |
| 2020-Sep | Vegetated | Deep | *H. crassispina* | Medium (4-5.9 cm) | Crevice | 10.19 | 3.14 | 17.98 |
| 2020-Sep | Vegetated | Shallow | *D. setosum* | Medium (4-5.9 cm) | Crevice | 13.26 | 2.78 | 25.52 |
| 2020-Sep | Vegetated | Shallow | *H. crassispina* | Medium (4-5.9 cm) | Pit | 8.20 | 1.30 | 16.31 |
| 2020-Sep | Vegetated | Shallow | *H. crassispina* | Medium (4-5.9 cm) | Crevice | 72.00 | 59.02 | 84.68 |
| 2020-Oct | Isoyake | Deep | *D. setosum* | Small (< 4 cm) | Crevice | 37.77 | 16.64 | 59.79 |
| 2020-Oct | Isoyake | Deep | *D. setosum* | Large (> 6 cm) | Free-living | 43.06 | 20.85 | 64.93 |
| 2020-Oct | Isoyake | Deep | *H. crassispina* | Medium (4-5.9 cm) | Crevice | 9.60 | 2.91 | 17.17 |
| 2020-Oct | Isoyake | Shallow | *D. savignyi* | Medium (4-5.9 cm) | Free-living | 9.85 | 2.14 | 18.27 |
| 2020-Oct | Isoyake | Shallow | *D. setosum* | Medium (4-5.9 cm) | Free-living | 44.09 | 28.77 | 58.94 |
| 2020-Oct | Isoyake | Shallow | *H. crassispina* | Large (> 6 cm) | Pit | 7.06 | 0.79 | 16.19 |
| 2020-Oct | Isoyake | Shallow | *H. crassispina* | Medium (4-5.9 cm) | Crevice | 22.58 | 12.31 | 33.09 |
| 2020-Oct | Isoyake | Shallow | *H. crassispina* | Medium (4-5.9 cm) | Free-living | 11.67 | 3.83 | 21.52 |
| 2020-Oct | Vegetated | Deep | *D. savignyi* | Medium (4-5.9 cm) | Crevice | 8.10 | 1.36 | 16.40 |
| 2020-Oct | Vegetated | Deep | *D. setosum* | Large (> 6 cm) | Crevice | 58.39 | 43.15 | 74.37 |
| 2020-Oct | Vegetated | Deep | *D. setosum* | Large (> 6 cm) | Free-living | 43.65 | 23.55 | 64.29 |
| 2020-Oct | Vegetated | Shallow | *D. savignyi* | Small (< 4 cm) | Pit | 3.99 | 0.25 | 9.53 |
| 2020-Oct | Vegetated | Shallow | *D. savignyi* | Medium (4-5.9 cm) | Crevice | 11.17 | 3.70 | 19.94 |
| 2020-Oct | Vegetated | Shallow | *D. setosum* | Small (< 4 cm) | Pit | 6.40 | 1.05 | 13.07 |
| 2020-Oct | Vegetated | Shallow | *D. setosum* | Large (> 6 cm) | Crevice | 13.41 | 3.09 | 25.32 |
| 2020-Oct | Vegetated | Shallow | *H. crassispina* | Small (< 4 cm) | Pit | 10.47 | 2.24 | 19.05 |
| 2020-Oct | Vegetated | Shallow | *H. crassispina* | Medium (4-5.9 cm) | Crevice | 70.50 | 58.16 | 82.24 |
| 2020-Nov | Isoyake | Deep | *D. setosum* | Medium (4-5.9 cm) | Crevice | 29.68 | 14.33 | 45.29 |
| 2020-Nov | Isoyake | Deep | *D. setosum* | Medium (4-5.9 cm) | Free-living | 65.09 | 50.35 | 79.17 |
| 2020-Nov | Isoyake | Deep | *H. crassispina* | Small (< 4 cm) | Crevice | 11.57 | 1.49 | 24.53 |
| 2020-Nov | Isoyake | Shallow | *D. savignyi* | Medium (4-5.9 cm) | Free-living | 10.26 | 2.40 | 18.77 |
| 2020-Nov | Isoyake | Shallow | *D. setosum* | Small (< 4 cm) | Pit | 10.79 | 2.88 | 19.86 |
| 2020-Nov | Isoyake | Shallow | *D. setosum* | Small (< 4 cm) | Crevice | 16.85 | 4.41 | 30.85 |
| 2020-Nov | Isoyake | Shallow | *D. setosum* | Medium (4-5.9 cm) | Free-living | 33.35 | 20.19 | 47.71 |
| 2020-Nov | Isoyake | Shallow | *H. crassispina* | Medium (4-5.9 cm) | Crevice | 22.67 | 12.49 | 33.08 |
| 2020-Nov | Vegetated | Deep | *D. savignyi* | Large (> 6 cm) | Crevice | 9.91 | 2.97 | 17.71 |
| 2020-Nov | Vegetated | Deep | *D. setosum* | Medium (4-5.9 cm) | Crevice | 73.07 | 57.23 | 87.34 |
| 2020-Nov | Vegetated | Deep | *D. setosum* | Medium (4-5.9 cm) | Free-living | 24.28 | 10.20 | 41.05 |
| 2020-Nov | Vegetated | Deep | *H. crassispina* | Medium (4-5.9 cm) | Crevice | 11.57 | 5.30 | 18.95 |
| 2020-Nov | Vegetated | Shallow | *D. savignyi* | Medium (4-5.9 cm) | Crevice | 11.76 | 3.63 | 20.80 |
| 2020-Nov | Vegetated | Shallow | *D. setosum* | Small (< 4 cm) | Pit | 7.28 | 0.96 | 15.50 |
| 2020-Nov | Vegetated | Shallow | *D. setosum* | Small (< 4 cm) | Crevice | 19.98 | 7.16 | 34.78 |
| 2020-Nov | Vegetated | Shallow | *H. crassispina* | Small (< 4 cm) | Pit | 13.16 | 4.01 | 24.35 |
| 2020-Nov | Vegetated | Shallow | *H. crassispina* | Medium (4-5.9 cm) | Crevice | 70.40 | 57.39 | 81.98 |
| 2020-Dec | Isoyake | Deep | *D. setosum* | Large (> 6 cm) | Crevice | 22.25 | 8.56 | 36.24 |
| 2020-Dec | Isoyake | Deep | *D. setosum* | Medium (4-5.9 cm) | Free-living | 50.55 | 34.38 | 66.51 |
| 2020-Dec | Isoyake | Deep | *H. crassispina* | Medium (4-5.9 cm) | Crevice | 11.80 | 4.50 | 20.51 |
| 2020-Dec | Isoyake | Shallow | *D. setosum* | Small (< 4 cm) | Pit | 11.06 | 2.83 | 20.94 |
| 2020-Dec | Isoyake | Shallow | *D. setosum* | Medium (4-5.9 cm) | Crevice | 24.04 | 9.58 | 39.89 |
| 2020-Dec | Isoyake | Shallow | *D. setosum* | Medium (4-5.9 cm) | Free-living | 24.40 | 12.64 | 36.53 |
| 2020-Dec | Isoyake | Shallow | *H. crassispina* | Medium (4-5.9 cm) | Pit | 10.71 | 3.56 | 19.04 |
| 2020-Dec | Isoyake | Shallow | *H. crassispina* | Medium (4-5.9 cm) | Crevice | 23.09 | 13.03 | 33.78 |
| 2020-Dec | Isoyake | Shallow | *H. crassispina* | Medium (4-5.9 cm) | Free-living | 12.88 | 4.68 | 22.65 |
| 2020-Dec | Vegetated | Deep | *D. setosum* | Large (> 6 cm) | Crevice | 72.07 | 57.37 | 85.52 |
| 2020-Dec | Vegetated | Deep | *H. crassispina* | Medium (4-5.9 cm) | Crevice | 12.09 | 5.42 | 19.31 |
| 2020-Dec | Vegetated | Shallow | *D. savignyi* | Large (> 6 cm) | Crevice | 11.41 | 4.28 | 19.83 |
| 2020-Dec | Vegetated | Shallow | *D. setosum* | Small (< 4 cm) | Crevice | 21.25 | 7.36 | 37.16 |
| 2020-Dec | Vegetated | Shallow | *H. crassispina* | Medium (4-5.9 cm) | Pit | 11.44 | 2.49 | 23.18 |
| 2020-Dec | Vegetated | Shallow | *H. crassispina* | Medium (4-5.9 cm) | Crevice | 69.68 | 56.88 | 82.11 |
| 2021-Feb | Isoyake | Deep | *D. savignyi* | Small (< 4 cm) | Crevice | 13.78 | 3.46 | 24.82 |
| 2021-Feb | Isoyake | Deep | *D. savignyi* | Medium (4-5.9 cm) | Free-living | 14.28 | 4.68 | 25.14 |
| 2021-Feb | Isoyake | Deep | *D. setosum* | Medium (4-5.9 cm) | Crevice | 37.59 | 20.78 | 53.77 |
| 2021-Feb | Isoyake | Deep | *D. setosum* | Medium (4-5.9 cm) | Free-living | 39.20 | 24.78 | 54.11 |
| 2021-Feb | Isoyake | Deep | *H. crassispina* | Medium (4-5.9 cm) | Crevice | 13.68 | 5.21 | 23.02 |
| 2021-Feb | Isoyake | Shallow | *D. savignyi* | Small (< 4 cm) | Pit | 5.54 | 0.90 | 11.70 |
| 2021-Feb | Isoyake | Shallow | *D. savignyi* | Medium (4-5.9 cm) | Crevice | 10.37 | 3.02 | 18.37 |
| 2021-Feb | Isoyake | Shallow | *D. setosum* | Medium (4-5.9 cm) | Crevice | 28.11 | 10.83 | 45.77 |
| 2021-Feb | Isoyake | Shallow | *D. setosum* | Medium (4-5.9 cm) | Free-living | 24.04 | 12.58 | 37.48 |
| 2021-Feb | Isoyake | Shallow | *H. crassispina* | Medium (4-5.9 cm) | Pit | 10.43 | 3.34 | 18.54 |
| 2021-Feb | Isoyake | Shallow | *H. crassispina* | Medium (4-5.9 cm) | Crevice | 23.25 | 13.53 | 33.08 |
| 2021-Feb | Isoyake | Shallow | *H. crassispina* | Medium (4-5.9 cm) | Free-living | 13.36 | 5.80 | 22.64 |
| 2021-Feb | Vegetated | Deep | *D. savignyi* | Large (> 6 cm) | Crevice | 11.78 | 5.19 | 19.26 |
| 2021-Feb | Vegetated | Deep | *D. setosum* | Medium (4-5.9 cm) | Crevice | 67.39 | 51.92 | 81.95 |
| 2021-Feb | Vegetated | Deep | *D. setosum* | Medium (4-5.9 cm) | Free-living | 11.56 | 3.73 | 20.76 |
| 2021-Feb | Vegetated | Deep | *H. crassispina* | Medium (4-5.9 cm) | Crevice | 10.96 | 4.94 | 17.42 |
| 2021-Feb | Vegetated | Shallow | *D. savignyi* | Small (< 4 cm) | Pit | 7.30 | 0.88 | 16.01 |
| 2021-Feb | Vegetated | Shallow | *D. savignyi* | Large (> 6 cm) | Crevice | 11.09 | 4.61 | 18.40 |
| 2021-Feb | Vegetated | Shallow | *H. crassispina* | Medium (4-5.9 cm) | Crevice | 64.42 | 51.01 | 77.26 |
| 2021-Feb | Vegetated | Shallow | *H. crassispina* | Medium (4-5.9 cm) | Free-living | 7.79 | 1.07 | 17.06 |
| 2021-Mar | Isoyake | Deep | *D. savignyi* | Medium (4-5.9 cm) | Crevice | 12.23 | 5.03 | 20.47 |
| 2021-Mar | Isoyake | Deep | *D. savignyi* | Medium (4-5.9 cm) | Free-living | 15.76 | 4.77 | 28.25 |
| 2021-Mar | Isoyake | Deep | *D. setosum* | Medium (4-5.9 cm) | Crevice | 35.84 | 18.00 | 53.58 |
| 2021-Mar | Isoyake | Deep | *D. setosum* | Medium (4-5.9 cm) | Free-living | 44.34 | 28.30 | 60.79 |
| 2021-Mar | Isoyake | Deep | *H. crassispina* | Medium (4-5.9 cm) | Crevice | 14.59 | 5.12 | 24.09 |
| 2021-Mar | Isoyake | Shallow | *D. savignyi* | Medium (4-5.9 cm) | Crevice | 11.99 | 3.69 | 21.51 |
| 2021-Mar | Isoyake | Shallow | *D. setosum* | Small (< 4 cm) | Crevice | 21.94 | 10.91 | 33.74 |
| 2021-Mar | Isoyake | Shallow | *D. setosum* | Medium (4-5.9 cm) | Free-living | 32.15 | 16.17 | 48.45 |
| 2021-Mar | Isoyake | Shallow | *H. crassispina* | Medium (4-5.9 cm) | Crevice | 23.71 | 14.23 | 33.91 |
| 2021-Mar | Isoyake | Shallow | *H. crassispina* | Medium (4-5.9 cm) | Free-living | 14.05 | 5.87 | 23.42 |
| 2021-Mar | Vegetated | Deep | *D. savignyi* | Medium (4-5.9 cm) | Crevice | 10.60 | 3.50 | 18.46 |
| 2021-Mar | Vegetated | Deep | *D. savignyi* | Medium (4-5.9 cm) | Free-living | 8.41 | 1.61 | 16.59 |
| 2021-Mar | Vegetated | Deep | *D. setosum* | Large (> 6 cm) | Crevice | 59.40 | 42.40 | 75.98 |
| 2021-Mar | Vegetated | Deep | *D. setosum* | Large (> 6 cm) | Free-living | 17.68 | 8.27 | 28.11 |
| 2021-Mar | Vegetated | Deep | *H. crassispina* | Medium (4-5.9 cm) | Crevice | 10.16 | 4.47 | 16.53 |
| 2021-Mar | Vegetated | Deep | *H. crassispina* | Large (> 6 cm) | Free-living | 11.34 | 1.32 | 24.92 |
| 2021-Mar | Vegetated | Shallow | *D. savignyi* | Large (> 6 cm) | Crevice | 11.08 | 4.83 | 18.00 |
| 2021-Mar | Vegetated | Shallow | *D. setosum* | Medium (4-5.9 cm) | Crevice | 13.16 | 5.25 | 22.02 |
| 2021-Mar | Vegetated | Shallow | *D. setosum* | Medium (4-5.9 cm) | Free-living | 1.96 | 0.17 | 4.76 |
| 2021-Mar | Vegetated | Shallow | *H. crassispina* | Small (< 4 cm) | Pit | 25.23 | 12.94 | 40.07 |
| 2021-Mar | Vegetated | Shallow | *H. crassispina* | Small (< 4 cm) | Crevice | 71.48 | 47.78 | 92.60 |
| 2021-Apr | Isoyake | Deep | *D. setosum* | Small (< 4 cm) | Crevice | 47.40 | 31.10 | 62.66 |
| 2021-Apr | Isoyake | Deep | *D. setosum* | Medium (4-5.9 cm) | Free-living | 51.71 | 34.81 | 68.98 |
| 2021-Apr | Isoyake | Deep | *H. crassispina* | Medium (4-5.9 cm) | Crevice | 15.34 | 5.13 | 25.47 |
| 2021-Apr | Isoyake | Shallow | *D. savignyi* | Large (> 6 cm) | Crevice | 7.51 | 2.17 | 14.60 |
| 2021-Apr | Isoyake | Shallow | *D. savignyi* | Medium (4-5.9 cm) | Free-living | 14.20 | 4.74 | 25.28 |
| 2021-Apr | Isoyake | Shallow | *D. setosum* | Small (< 4 cm) | Crevice | 20.75 | 10.40 | 31.23 |
| 2021-Apr | Isoyake | Shallow | *D. setosum* | Medium (4-5.9 cm) | Free-living | 41.85 | 22.81 | 61.91 |
| 2021-Apr | Isoyake | Shallow | *H. crassispina* | Medium (4-5.9 cm) | Crevice | 24.35 | 14.42 | 35.69 |
| 2021-Apr | Isoyake | Shallow | *H. crassispina* | Medium (4-5.9 cm) | Free-living | 14.32 | 5.78 | 23.78 |
| 2021-Apr | Vegetated | Deep | *D. savignyi* | Large (> 6 cm) | Crevice | 13.08 | 6.34 | 20.47 |
| 2021-Apr | Vegetated | Deep | *D. savignyi* | Medium (4-5.9 cm) | Free-living | 10.12 | 2.38 | 19.61 |
| 2021-Apr | Vegetated | Deep | *D. setosum* | Large (> 6 cm) | Crevice | 52.02 | 31.95 | 70.89 |
| 2021-Apr | Vegetated | Deep | *D. setosum* | Large (> 6 cm) | Free-living | 22.98 | 10.97 | 35.75 |
| 2021-Apr | Vegetated | Deep | *H. crassispina* | Medium (4-5.9 cm) | Crevice | 9.29 | 3.55 | 15.22 |
| 2021-Apr | Vegetated | Shallow | *D. savignyi* | Medium (4-5.9 cm) | Crevice | 11.28 | 3.05 | 20.65 |
| 2021-Apr | Vegetated | Shallow | *D. setosum* | Small (< 4 cm) | Crevice | 11.36 | 2.47 | 22.19 |
| 2021-Apr | Vegetated | Shallow | *H. crassispina* | Small (< 4 cm) | Pit | 28.01 | 13.74 | 43.49 |
| 2021-Apr | Vegetated | Shallow | *H. crassispina* | Medium (4-5.9 cm) | Crevice | 58.69 | 45.60 | 72.05 |
| 2021-May | Isoyake | Deep | *D. savignyi* | Medium (4-5.9 cm) | Crevice | 16.42 | 6.60 | 27.15 |
| 2021-May | Isoyake | Deep | *D. setosum* | Small (< 4 cm) | Crevice | 40.43 | 24.07 | 57.04 |
| 2021-May | Isoyake | Deep | *D. setosum* | Large (> 6 cm) | Free-living | 23.54 | 8.54 | 40.53 |
| 2021-May | Isoyake | Deep | *H. crassispina* | Medium (4-5.9 cm) | Crevice | 14.86 | 4.89 | 25.75 |
| 2021-May | Isoyake | Shallow | *D. setosum* | Small (< 4 cm) | Pit | 11.47 | 3.59 | 20.90 |
| 2021-May | Isoyake | Shallow | *D. setosum* | Small (< 4 cm) | Crevice | 17.65 | 8.25 | 27.10 |
| 2021-May | Isoyake | Shallow | *H. crassispina* | Small (< 4 cm) | Pit | 10.44 | 1.87 | 21.13 |
| 2021-May | Isoyake | Shallow | *H. crassispina* | Medium (4-5.9 cm) | Crevice | 23.39 | 12.97 | 34.45 |
| 2021-May | Vegetated | Deep | *D. savignyi* | Large (> 6 cm) | Crevice | 13.03 | 6.30 | 20.58 |
| 2021-May | Vegetated | Deep | *D. setosum* | Medium (4-5.9 cm) | Crevice | 43.96 | 29.71 | 57.95 |
| 2021-May | Vegetated | Deep | *D. setosum* | Large (> 6 cm) | Free-living | 30.75 | 15.04 | 47.19 |
| 2021-May | Vegetated | Deep | *H. crassispina* | Medium (4-5.9 cm) | Crevice | 7.85 | 2.91 | 13.36 |
| 2021-May | Vegetated | Shallow | *D. savignyi* | Medium (4-5.9 cm) | Crevice | 10.83 | 2.88 | 20.50 |
| 2021-May | Vegetated | Shallow | *D. setosum* | Medium (4-5.9 cm) | Crevice | 8.86 | 3.53 | 15.25 |
| 2021-May | Vegetated | Shallow | *H. crassispina* | Medium (4-5.9 cm) | Crevice | 54.35 | 40.29 | 67.48 |
| 2021-Jun | Isoyake | Deep | *D. savignyi* | Medium (4-5.9 cm) | Crevice | 16.91 | 5.93 | 29.61 |
| 2021-Jun | Isoyake | Deep | *D. setosum* | Small (< 4 cm) | Pit | 15.51 | 2.86 | 30.25 |
| 2021-Jun | Isoyake | Deep | *D. setosum* | Small (< 4 cm) | Crevice | 32.59 | 14.15 | 51.02 |
| 2021-Jun | Isoyake | Deep | *D. setosum* | Medium (4-5.9 cm) | Free-living | 70.77 | 53.54 | 86.53 |
| 2021-Jun | Isoyake | Deep | *H. crassispina* | Medium (4-5.9 cm) | Crevice | 13.64 | 3.89 | 25.26 |
| 2021-Jun | Isoyake | Shallow | *D. savignyi* | Small (< 4 cm) | Free-living | 10.85 | 2.98 | 20.15 |
| 2021-Jun | Isoyake | Shallow | *D. setosum* | Small (< 4 cm) | Pit | 10.21 | 2.28 | 20.37 |
| 2021-Jun | Isoyake | Shallow | *D. setosum* | Small (< 4 cm) | Crevice | 13.88 | 5.52 | 23.39 |
| 2021-Jun | Isoyake | Shallow | *D. setosum* | Small (< 4 cm) | Free-living | 23.05 | 8.38 | 38.30 |
| 2021-Jun | Isoyake | Shallow | *H. crassispina* | Medium (4-5.9 cm) | Pit | 10.65 | 3.86 | 18.04 |
| 2021-Jun | Isoyake | Shallow | *H. crassispina* | Medium (4-5.9 cm) | Crevice | 21.41 | 10.44 | 31.82 |
| 2021-Jun | Isoyake | Shallow | *H. crassispina* | Medium (4-5.9 cm) | Free-living | 12.36 | 4.56 | 21.15 |
| 2021-Jun | Vegetated | Deep | *D. savignyi* | Medium (4-5.9 cm) | Crevice | 10.37 | 3.73 | 18.39 |
| 2021-Jun | Vegetated | Deep | *D. savignyi* | Large (> 6 cm) | Free-living | 13.25 | 4.63 | 23.99 |
| 2021-Jun | Vegetated | Deep | *D. setosum* | Medium (4-5.9 cm) | Crevice | 36.54 | 23.83 | 50.17 |
| 2021-Jun | Vegetated | Deep | *D. setosum* | Medium (4-5.9 cm) | Free-living | 35.11 | 17.88 | 51.48 |
| 2021-Jun | Vegetated | Deep | *H. crassispina* | Medium (4-5.9 cm) | Crevice | 6.61 | 2.19 | 11.64 |
| 2021-Jun | Vegetated | Shallow | *D. savignyi* | Large (> 6 cm) | Crevice | 10.52 | 4.09 | 17.44 |
| 2021-Jun | Vegetated | Shallow | *D. savignyi* | Large (> 6 cm) | Free-living | 8.39 | 2.67 | 15.20 |
| 2021-Jun | Vegetated | Shallow | *D. setosum* | Medium (4-5.9 cm) | Pit | 6.76 | 0.49 | 16.09 |
| 2021-Jun | Vegetated | Shallow | *D. setosum* | Medium (4-5.9 cm) | Crevice | 7.25 | 2.37 | 12.70 |
| 2021-Jun | Vegetated | Shallow | *D. setosum* | Large (> 6 cm) | Free-living | 13.50 | 3.34 | 24.66 |
| 2021-Jun | Vegetated | Shallow | *H. crassispina* | Small (< 4 cm) | Pit | 24.10 | 12.68 | 36.26 |
| 2021-Jun | Vegetated | Shallow | *H. crassispina* | Medium (4-5.9 cm) | Crevice | 50.29 | 35.26 | 64.14 |
| 2021-Jul | Isoyake | Deep | *D. savignyi* | Small (< 4 cm) | Crevice | 29.97 | 12.73 | 48.12 |
| 2021-Jul | Isoyake | Deep | *D. setosum* | Small (< 4 cm) | Free-living | 55.33 | 29.91 | 79.98 |
| 2021-Jul | Isoyake | Shallow | *D. savignyi* | Small (< 4 cm) | Free-living | 9.93 | 2.05 | 19.42 |
| 2021-Jul | Isoyake | Shallow | *D. setosum* | Medium (4-5.9 cm) | Pit | 21.60 | 3.90 | 44.31 |
| 2021-Jul | Isoyake | Shallow | *D. setosum* | Small (< 4 cm) | Crevice | 10.90 | 2.77 | 20.12 |
| 2021-Jul | Isoyake | Shallow | *D. setosum* | Small (< 4 cm) | Free-living | 24.22 | 8.30 | 42.11 |
| 2021-Jul | Isoyake | Shallow | *H. crassispina* | Medium (4-5.9 cm) | Pit | 9.92 | 3.97 | 17.16 |
| 2021-Jul | Isoyake | Shallow | *H. crassispina* | Medium (4-5.9 cm) | Crevice | 20.16 | 10.04 | 31.06 |
| 2021-Jul | Isoyake | Shallow | *H. crassispina* | Medium (4-5.9 cm) | Free-living | 11.58 | 3.28 | 21.15 |
| 2021-Jul | Vegetated | Deep | *D. savignyi* | Medium (4-5.9 cm) | Crevice | 10.02 | 3.50 | 17.90 |
| 2021-Jul | Vegetated | Deep | *D. savignyi* | Medium (4-5.9 cm) | Free-living | 11.43 | 3.84 | 20.35 |
| 2021-Jul | Vegetated | Deep | *D. setosum* | Medium (4-5.9 cm) | Crevice | 33.36 | 20.34 | 46.45 |
| 2021-Jul | Vegetated | Deep | *D. setosum* | Medium (4-5.9 cm) | Free-living | 41.28 | 23.59 | 59.63 |
| 2021-Jul | Vegetated | Deep | *H. crassispina* | Large (> 6 cm) | Crevice | 11.46 | 3.73 | 19.62 |
| 2021-Jul | Vegetated | Shallow | *D. savignyi* | Medium (4-5.9 cm) | Crevice | 10.10 | 2.89 | 19.29 |
| 2021-Jul | Vegetated | Shallow | *D. savignyi* | Large (> 6 cm) | Free-living | 8.01 | 2.15 | 14.58 |
| 2021-Jul | Vegetated | Shallow | *D. setosum* | Medium (4-5.9 cm) | Crevice | 6.78 | 2.20 | 11.95 |
| 2021-Jul | Vegetated | Shallow | *D. setosum* | Medium (4-5.9 cm) | Free-living | 9.10 | 2.25 | 16.95 |
| 2021-Jul | Vegetated | Shallow | *H. crassispina* | Small (< 4 cm) | Pit | 20.15 | 10.09 | 30.71 |
| 2021-Jul | Vegetated | Shallow | *H. crassispina* | Medium (4-5.9 cm) | Crevice | 49.79 | 34.49 | 64.30 |
| 2021-Aug | Isoyake | Deep | *D. savignyi* | Small (< 4 cm) | Crevice | 26.81 | 8.67 | 46.82 |
| 2021-Aug | Isoyake | Deep | *D. savignyi* | Small (< 4 cm) | Free-living | 21.43 | 6.78 | 36.54 |
| 2021-Aug | Isoyake | Shallow | *D. savignyi* | Medium (4-5.9 cm) | Pit | 9.04 | 1.48 | 20.09 |
| 2021-Aug | Isoyake | Shallow | *D. savignyi* | Small (< 4 cm) | Crevice | 10.44 | 3.51 | 18.57 |
| 2021-Aug | Isoyake | Shallow | *D. savignyi* | Medium (4-5.9 cm) | Free-living | 13.57 | 2.96 | 25.44 |
| 2021-Aug | Isoyake | Shallow | *D. setosum* | Small (< 4 cm) | Crevice | 9.50 | 1.42 | 20.06 |
| 2021-Aug | Isoyake | Shallow | *D. setosum* | Medium (4-5.9 cm) | Free-living | 72.56 | 58.87 | 84.55 |
| 2021-Aug | Isoyake | Shallow | *H. crassispina* | Medium (4-5.9 cm) | Pit | 9.70 | 3.25 | 16.65 |
| 2021-Aug | Isoyake | Shallow | *H. crassispina* | Small (< 4 cm) | Crevice | 11.27 | 1.42 | 24.29 |
| 2021-Aug | Vegetated | Deep | *D. savignyi* | Large (> 6 cm) | Crevice | 11.76 | 5.11 | 19.50 |
| 2021-Aug | Vegetated | Deep | *D. savignyi* | Medium (4-5.9 cm) | Free-living | 9.25 | 2.36 | 17.21 |
| 2021-Aug | Vegetated | Deep | *D. setosum* | Medium (4-5.9 cm) | Crevice | 35.63 | 23.61 | 49.10 |
| 2021-Aug | Vegetated | Deep | *D. setosum* | Large (> 6 cm) | Free-living | 44.51 | 18.84 | 69.09 |
| 2021-Aug | Vegetated | Deep | *H. crassispina* | Large (> 6 cm) | Crevice | 11.55 | 3.62 | 20.63 |
| 2021-Aug | Vegetated | Shallow | *D. savignyi* | Large (> 6 cm) | Crevice | 10.60 | 3.96 | 17.50 |
| 2021-Aug | Vegetated | Shallow | *D. savignyi* | Medium (4-5.9 cm) | Free-living | 5.50 | 0.49 | 12.45 |
| 2021-Aug | Vegetated | Shallow | *D. setosum* | Large (> 6 cm) | Crevice | 8.16 | 1.19 | 17.38 |
| 2021-Aug | Vegetated | Shallow | *D. setosum* | Large (> 6 cm) | Free-living | 16.95 | 3.01 | 35.02 |
| 2021-Aug | Vegetated | Shallow | *H. crassispina* | Small (< 4 cm) | Pit | 16.91 | 6.86 | 27.08 |
| 2021-Aug | Vegetated | Shallow | *H. crassispina* | Medium (4-5.9 cm) | Crevice | 54.69 | 40.16 | 68.65 |
| 2021-Aug | Vegetated | Shallow | *H. crassispina* | Medium (4-5.9 cm) | Free-living | 6.51 | 0.86 | 13.49 |
| 2021-Sep | Isoyake | Deep | *D. savignyi* | Small (< 4 cm) | Free-living | 17.25 | 2.25 | 35.08 |
| 2021-Sep | Isoyake | Deep | *D. setosum* | Medium (4-5.9 cm) | Free-living | 77.00 | 66.30 | 88.01 |
| 2021-Sep | Isoyake | Deep | *H. crassispina* | Medium (4-5.9 cm) | Free-living | 10.86 | 0.71 | 24.25 |
| 2021-Sep | Isoyake | Shallow | *D. savignyi* | Large (> 6 cm) | Free-living | 6.68 | 0.94 | 14.21 |
| 2021-Sep | Isoyake | Shallow | *D. setosum* | Medium (4-5.9 cm) | Free-living | 71.82 | 55.48 | 86.12 |
| 2021-Sep | Isoyake | Shallow | *H. crassispina* | Medium (4-5.9 cm) | Pit | 10.16 | 3.29 | 17.97 |
| 2021-Sep | Isoyake | Shallow | *H. crassispina* | Medium (4-5.9 cm) | Crevice | 25.85 | 13.87 | 38.43 |
| 2021-Sep | Vegetated | Deep | *D. savignyi* | Medium (4-5.9 cm) | Crevice | 10.35 | 3.29 | 17.98 |
| 2021-Sep | Vegetated | Deep | *D. savignyi* | Medium (4-5.9 cm) | Free-living | 7.25 | 0.70 | 15.48 |
| 2021-Sep | Vegetated | Deep | *D. setosum* | Medium (4-5.9 cm) | Crevice | 41.19 | 27.80 | 54.86 |
| 2021-Sep | Vegetated | Deep | *D. setosum* | Medium (4-5.9 cm) | Free-living | 34.82 | 21.68 | 48.47 |
| 2021-Sep | Vegetated | Deep | *H. crassispina* | Medium (4-5.9 cm) | Crevice | 8.15 | 3.28 | 13.71 |
| 2021-Sep | Vegetated | Shallow | *D. savignyi* | Large (> 6 cm) | Crevice | 11.20 | 4.42 | 19.10 |
| 2021-Sep | Vegetated | Shallow | *D. savignyi* | Large (> 6 cm) | Free-living | 6.45 | 0.50 | 14.06 |
| 2021-Sep | Vegetated | Shallow | *D. setosum* | Medium (4-5.9 cm) | Crevice | 10.58 | 4.35 | 16.83 |
| 2021-Sep | Vegetated | Shallow | *D. setosum* | Medium (4-5.9 cm) | Free-living | 8.06 | 1.06 | 16.70 |
| 2021-Sep | Vegetated | Shallow | *H. crassispina* | Small (< 4 cm) | Pit | 14.67 | 4.12 | 27.04 |
| 2021-Sep | Vegetated | Shallow | *H. crassispina* | Medium (4-5.9 cm) | Crevice | 62.90 | 49.55 | 76.05 |
| 2021-Sep | Vegetated | Shallow | *H. crassispina* | Medium (4-5.9 cm) | Free-living | 5.76 | 0.36 | 13.68 |
| 2021-Oct | Isoyake | Deep | *D. setosum* | Medium (4-5.9 cm) | Crevice | 22.07 | 11.15 | 33.69 |
| 2021-Oct | Isoyake | Deep | *D. setosum* | Medium (4-5.9 cm) | Free-living | 76.70 | 64.21 | 88.15 |
| 2021-Oct | Isoyake | Shallow | *D. savignyi* | Small (< 4 cm) | Crevice | 10.38 | 1.34 | 22.64 |
| 2021-Oct | Isoyake | Shallow | *D. setosum* | Medium (4-5.9 cm) | Pit | 24.42 | 1.80 | 58.56 |
| 2021-Oct | Isoyake | Shallow | *D. setosum* | Medium (4-5.9 cm) | Crevice | 27.12 | 13.61 | 41.36 |
| 2021-Oct | Isoyake | Shallow | *D. setosum* | Medium (4-5.9 cm) | Free-living | 72.28 | 50.91 | 91.15 |
| 2021-Oct | Isoyake | Shallow | *H. crassispina* | Medium (4-5.9 cm) | Pit | 11.23 | 3.05 | 20.56 |
| 2021-Oct | Isoyake | Shallow | *H. crassispina* | Medium (4-5.9 cm) | Crevice | 32.56 | 17.60 | 48.11 |
| 2021-Oct | Vegetated | Deep | *D. savignyi* | Large (> 6 cm) | Crevice | 10.08 | 3.21 | 17.66 |
| 2021-Oct | Vegetated | Deep | *D. setosum* | Medium (4-5.9 cm) | Crevice | 45.31 | 29.01 | 60.36 |
| 2021-Oct | Vegetated | Deep | *D. setosum* | Medium (4-5.9 cm) | Free-living | 32.14 | 17.08 | 46.51 |
| 2021-Oct | Vegetated | Deep | *H. crassispina* | Medium (4-5.9 cm) | Crevice | 9.88 | 3.58 | 17.07 |
| 2021-Oct | Vegetated | Shallow | *D. savignyi* | Medium (4-5.9 cm) | Crevice | 15.44 | 4.01 | 29.36 |
| 2021-Oct | Vegetated | Shallow | *D. setosum* | Medium (4-5.9 cm) | Crevice | 13.68 | 5.87 | 22.74 |
| 2021-Oct | Vegetated | Shallow | *H. crassispina* | Small (< 4 cm) | Pit | 13.26 | 1.53 | 28.13 |
| 2021-Oct | Vegetated | Shallow | *H. crassispina* | Medium (4-5.9 cm) | Crevice | 70.67 | 57.14 | 83.90 |
| 2021-Nov | Isoyake | Deep | *D. setosum* | Medium (4-5.9 cm) | Crevice | 21.32 | 8.64 | 35.40 |
| 2021-Nov | Isoyake | Deep | *D. setosum* | Medium (4-5.9 cm) | Free-living | 78.42 | 63.18 | 91.91 |
| 2021-Nov | Isoyake | Deep | *H. crassispina* | Medium (4-5.9 cm) | Crevice | 18.00 | 1.84 | 40.26 |
| 2021-Nov | Isoyake | Shallow | *D. savignyi* | Medium (4-5.9 cm) | Crevice | 18.38 | 2.15 | 37.91 |
| 2021-Nov | Isoyake | Shallow | *D. setosum* | Medium (4-5.9 cm) | Crevice | 29.29 | 12.21 | 46.10 |
| 2021-Nov | Isoyake | Shallow | *D. setosum* | Small (< 4 cm) | Free-living | 13.74 | 0.18 | 31.62 |
| 2021-Nov | Isoyake | Shallow | *H. crassispina* | Medium (4-5.9 cm) | Crevice | 40.09 | 21.00 | 60.99 |
| 2021-Nov | Vegetated | Deep | *D. savignyi* | Large (> 6 cm) | Crevice | 9.58 | 2.15 | 19.00 |
| 2021-Nov | Vegetated | Deep | *D. setosum* | Large (> 6 cm) | Pit | 6.55 | 0.14 | 16.50 |
| 2021-Nov | Vegetated | Deep | *D. setosum* | Medium (4-5.9 cm) | Crevice | 46.22 | 26.25 | 64.30 |
| 2021-Nov | Vegetated | Deep | *D. setosum* | Medium (4-5.9 cm) | Free-living | 32.92 | 14.60 | 52.06 |
| 2021-Nov | Vegetated | Deep | *H. crassispina* | Medium (4-5.9 cm) | Crevice | 11.64 | 3.51 | 21.71 |
| 2021-Nov | Vegetated | Shallow | *D. savignyi* | Large (> 6 cm) | Crevice | 13.35 | 2.47 | 25.35 |
| 2021-Nov | Vegetated | Shallow | *D. setosum* | Medium (4-5.9 cm) | Crevice | 16.03 | 5.63 | 27.92 |
| 2021-Nov | Vegetated | Shallow | *H. crassispina* | Medium (4-5.9 cm) | Pit | 9.59 | 0.54 | 21.91 |
| 2021-Nov | Vegetated | Shallow | *H. crassispina* | Medium (4-5.9 cm) | Crevice | 76.51 | 61.95 | 90.44 |
| 2021-Dec | Isoyake | Deep | *D. savignyi* | Large (> 6 cm) | Free-living | 9.34 | 0.67 | 22.46 |
| 2021-Dec | Isoyake | Deep | *D. setosum* | Medium (4-5.9 cm) | Crevice | 20.76 | 4.28 | 39.64 |
| 2021-Dec | Isoyake | Deep | *D. setosum* | Medium (4-5.9 cm) | Free-living | 79.83 | 59.61 | 96.80 |
| 2021-Dec | Isoyake | Shallow | *D. setosum* | Medium (4-5.9 cm) | Crevice | 31.49 | 9.52 | 55.47 |
| 2021-Dec | Isoyake | Shallow | *H. crassispina* | Medium (4-5.9 cm) | Pit | 15.01 | 1.13 | 32.78 |
| 2021-Dec | Isoyake | Shallow | *H. crassispina* | Medium (4-5.9 cm) | Crevice | 47.83 | 21.10 | 74.65 |
| 2021-Dec | Vegetated | Deep | *D. savignyi* | Medium (4-5.9 cm) | Crevice | 12.17 | 1.05 | 26.90 |
| 2021-Dec | Vegetated | Deep | *D. setosum* | Medium (4-5.9 cm) | Crevice | 46.51 | 20.75 | 71.15 |
| 2021-Dec | Vegetated | Deep | *D. setosum* | Medium (4-5.9 cm) | Free-living | 34.79 | 9.05 | 61.10 |
| 2021-Dec | Vegetated | Deep | *H. crassispina* | Medium (4-5.9 cm) | Crevice | 13.90 | 2.34 | 29.00 |
| 2021-Dec | Vegetated | Shallow | *D. savignyi* | Large (> 6 cm) | Crevice | 15.14 | 1.41 | 33.34 |
| 2021-Dec | Vegetated | Shallow | *D. setosum* | Medium (4-5.9 cm) | Crevice | 18.74 | 4.08 | 37.05 |
| 2021-Dec | Vegetated | Shallow | *H. crassispina* | Medium (4-5.9 cm) | Crevice | 80.86 | 63.68 | 95.96 |
