# Supplementary material for: The behavior of sympatric sea urchin species across an ecosystem state gradient
Source: PeerJ. 2023 Jun 13;11:e15511. doi: 10.7717/peerj.15511 (PMC10274604; doi:10.7717/peerj.15511)
Supplement: Supplemental Information 11 — The mean and 95% highest density credible interval for the expectations of the generalized linear model (GLM) on the sea urchin linear displacement of the mark-recapture experiment in the isoyake and vegetated habitat. [file peerj-11-15511-s011.docx]

| **Habitat** | **Species** | **Survey** | **Linear displacement (m) GLM** | | |
| --- | --- | --- | --- | --- | --- |
|  |  |  | **Mean** | **Lower** | **Upper** |
| Isoyake | *D. setosum* | 1st recapture | 1.36 | 0.74 | 2.07 |
| Isoyake | *D. setosum* | 2nd recapture | 3.15 | 1.81 | 4.68 |
| Isoyake | *H. crassispina* | 1st recapture | 0.07 | 0.01 | 0.16 |
| Isoyake | *H. crassispina* | 2nd recapture | 0.50 | 0.28 | 0.74 |
| Vegetated | *D. setosum* | 1st recapture | 0.97 | 0.56 | 1.42 |
| Vegetated | *D. setosum* | 2nd recapture | 1.95 | 1.16 | 2.82 |
| Vegetated | *H. crassispina* | 1st recapture | 0.02 | 0.00 | 0.09 |
| Vegetated | *H. crassispina* | 2nd recapture | 0.09 | 0.00 | 0.35 |
