# Supplementary material for: The behavior of sympatric sea urchin species across an ecosystem state gradient
Source: PeerJ. 2023 Jun 13;11:e15511. doi: 10.7717/peerj.15511 (PMC10274604; doi:10.7717/peerj.15511)
Supplement: Supplemental Information 12 — The mean and 95% highest density credible interval for the expectations of the model on the sea urchin group composition of the mark-recapture experiment in the isoyake and vegetated habitat. [file peerj-11-15511-s012.docx]

| **Habitat** | **Species** | **Survey** | **Group-size (indiv.) GLM** | | |
| --- | --- | --- | --- | --- | --- |
|  |  |  | **Mean** | **Lower** | **Upper** |
| Isoyake | *D. setosum* | start | 4.38 | 1.91 | 7.25 |
| Isoyake | *D. setosum* | 1st recapture | 2.16 | 0.57 | 4.11 |
| Isoyake | *D. setosum* | 2nd recapture | 5.29 | 1.51 | 10.00 |
| Vegetated | *D. setosum* | start | 7.73 | 1.72 | 16.45 |
| Vegetated | *D. setosum* | 1st recapture | 5.55 | 0.76 | 13.43 |
| Vegetated | *D. setosum* | 2nd recapture | 4.80 | 0.57 | 12.45 |
